# Supplementary material for: Financial implications of New York City’s weight management initiative
Source: PLoS One. 2021 Feb 11;16(2):e0246621. doi: 10.1371/journal.pone.0246621 (PMC7877753; doi:10.1371/journal.pone.0246621)
Supplement: S1 Table — (DOCX) [file pone.0246621.s001.docx]

**S1 Table. Net Savings**

| Net Savings | Digital | Workshops | Total |
| --- | --- | --- | --- |
| *Employees (N=12,436)* | | | |
| Total Net Savings | $327,882 | $1,158,220 | $1,486,102 |
| % of Total Participants | 44% | 56% | 100% |
| % of Total Net Savings | 22% | 78% | 100% |
| *Dependents (N=2,510)* | | | |
| Total Net Savings | $77,214 | $400,115 | $477,329 |
| % of Total Participants | 33% | 67% | 100% |
| % of Total Net Savings | 16% | 84% | 100% |
