# Supplementary material for: Financial implications of New York City’s weight management initiative
Source: PLoS One. 2021 Feb 11;16(2):e0246621. doi: 10.1371/journal.pone.0246621 (PMC7877753; doi:10.1371/journal.pone.0246621)
Supplement: S1 File — (DOCX) [file pone.0246621.s002.docx]

**S1Text. Steps of Analysis**

This is a supplement to the paper “Participation, Weight Loss, and Financial Implications of City of New York’s Weight Management Initiative”. The paper presents the rate of participation in a Weight Watchers program subsidized for employees by the City of New York Benefits Program and Unions. It also presents weight change among participants and applies the weight loss among program participants along with their starting BMI to the medical expenditure savings presented in Cawley et al. to simulate savings generated from participation in the WW program. This supplement describes the steps of analysis employed in the paper.

**Sample:**

Members *eligible* for analysis: All NYC beneficiaries (employees and dependents) with data who meet the following inclusion criteria

1. Are aged ≥ 18 and < 65 years at the end of the analysis year

- Costs savings cannot be claimed for those aged 65 and older. The majority of people aged 65+ are on Medicare and while there are active employees aged 65+ who have the City’s health plan as their primary insurance, they will be hard to identify

1. Have a baseline BMI ≥ 26 kg/m^2^ per the lower threshold of Cawley et al. model, with no upper limit
2. Enrolled during evaluation period (June 1, 2016 – May 31, 2017)
3. Activated WW membership between June 1, 2016- March 31, 2017 to allow for 2 months until end of evaluation period, May 31, 2017)

Out of 19,371 participants enrolled, the sample eligible for analysis in our paper consists of 14,946 participants.

Members *that are eligible to contribute to savings* in analysis: All eligible NYC beneficiaries who meet the following criteria:

1. Must have a starting weight and height
2. Enrollment height and weight are greater than the 1st percentile for NHANES 2011-2014 data, and physiologically plausible

- Height ≥ 58 inches and ≤ 84 inches
- Weight ≥ 104 lbs. and ≤ 700 lbs.
- Weight Watchers has many members above the 99th NHANES percentile in weight and height so upper limits are based on physiological plausibility

1. Weight change is physiologically feasible in 12 months

- Total weight loss ≤ 25%
- Total weight gain ≤ 25%
- Average weight loss per month ≤ 8.5%

These inclusion criteria mean that there will be a discrepancy between the number of WW members with NYC Weight Watchers program costs and the number of WW members with NYC cost-savings, and only a proportion of the total WW members with NYC costs will be contributing to savings.

Out of the sample of 14,946 participants, 1,980 participants have bad data (implausible height, weight or weight change) and do not contribute to savings. Further, 154 participants have only one weight recorded, becoming ineligible to contribute to savings in our analysis. Thus 12,812 participants, i.e. 86% of participants eligible for analysis, are eligible to contribute to savings in the analysis, while all employees (12,436 out of the 14,946 participants) contribute to costs. We present distribution of savings for only the 12,812 participants which we call the savings sample. 10,766 employees (87%) and 2,046 dependents (82%) constitute the savings sample.

**Analysis Steps:**

The steps for analyzing the fifteen months (June 1, 2016 – August 31, 2017) of data recorded are as follows:

1. Adjust Cawley et al. savings for inflation @22.37% for period 2010-2017.
2. Calculate % reduction in BMI [(last weight – baseline weight)/baseline weight*100]
3. Assign savings based on the inflation adjusted Cawley et al. table.
4. Calculate and apply adjustment based on participation duration (days out of 365).
5. Apply adjustment for the percentage (74.7%) of total medical expenditures that are covered by the CONY benefit plans^^[[1]](#footnote-1)^^. The savings are multiplied by 0.747.
6. Calculate NYC program costs. These costs are for employees only. Costs are calculated for duration of enrollment, which is the period between enrollment date and disenrollment date or end of evaluation period, whichever comes first. As long as an employee is enrolled for even one day of a month, we assign him cost for the entire month. Thus, we calculate enrollment duration (months rounded to the next integer) for employees and multiply this duration (in months engaged) by $15 for Workshops or $7 for Online.
7. Subtract NYC WW program costs from savings to get net savings.

**Examples of Analysis:**

Person A

1. Employee, Workshops
2. Age 45, starting BMI 33 kg/m^2^
3. Enrolled/Baseline weight: date= August 1, 2016, weight= 200 lbs
4. Final weight in Evaluation Year 1 (June 1, 2016-May 31, 2017): date= May 15, 2017, weight= 190 lbs
5. Disenrollment date= missing
6. Had a weight in the 3 months directly following Evaluation period: date= July 5, 2017, weight=180 lbs

Person B

1. Dependent, Online
2. Age 45, starting BMI 33 kg/m^2^
3. Enrolled/Baseline weight: date=August 1, 2016, weight= 200 lbs
4. Final weight in Evaluation period 1 (June 1, 2016-May 31, 2017): date= April 15, 2017, weight= 190 lbs
5. Disenrollment date= April 15, 2017
6. Did not have a weight in the 3 months directly following Evaluation period

Evaluation:

Person A:

1. Calculate % reduction in BMI [((last weight- baseline weight)/baseline weight)*100]
   1. Because they have a weight in the 2 months following the end of the Evaluation Year, their Last Weight is Weighted
      1. 51 days between May 15-July 5, 46 days from May 15-June 30, 5 days from June 30-July 5
      2. Weight from July 5 gets weighed 0.91 (46/51), weight from May 15 gets weighed 0.09 (5/51)
   2. Last weight= (180 lbs. * 0.91) + (190 lbs. * 0.09)= 163.8 + 17.1= 180.9
   3. % reduction in BMI = ((180.9 -200)/200)*100= (19.1/200)*100= 9.55%
2. Assign savings based on inflation adjusted Cawley et al. table. The reduction in BMI is rounded to the nearest integer. Hence, for a person with enrollment BMI 33 kg/m^2^ and % reduction in BMI = 10, inflation adjusted Cawley et al. table lists savings = $548.23
3. Calculate and apply adjustment based on participation duration
   1. Because they have a weight in the 2 months following the end of the evaluation period, their duration is the number of days between baseline date and last date of evaluation period (August 1, 2016-May 31, 2017)
   2. Engaged in Evaluation period for 304 days, adjust by 0.83 (304/365)
   3. $548.23* 0.83=$455.03
4. Apply adjustment for the percentage of total medical expenditures covered by CONY benefit plans
   1. 74.7% covered
   2. $455.03* 0.747= $339.91
5. Calculate NYC program costs
   1. Costs are for employees only, and are $15/month for Workshops
   2. Disenrollment date missing, hence assumed to dis-enroll at end of evaluation period i.e. on 31 May, 2017. Enrollment duration is 304 days is 10.1 thirty day months, therefore equivalent to 11 months of payments.
   3. 11 * $15= $165
6. Subtract NYC WW program costs from savings
   1. $339.91 - $165= $174.91

Person B:

1. Calculate % reduction in BMI [((last weight- baseline weight)/baseline weight)*100]
   1. Because they do not have a weight in the 2 months following the end of the Evaluation Year, their Last Weight is the latest weight in the Evaluation Year
   2. Last weight= 190
   3. % reduction in BMI = ((190 -200)/200)*100= (10/200)*100= 5%
2. Assign savings based on inflation adjusted Cawley et al. table. For a person with enrollment BMI of 33 and % reduction in BMI=5, inflation adjusted Cawley et al. table lists savings as = $352.81
3. Calculate and apply adjustment based on participation duration
   1. Because they do not have a weight in the 2 months following the end of the Evaluation, their duration is the number of days between baseline date and date of Last Weight (August 1, 2016-April 15, 2017)
   2. Engaged in Evaluation period for 257 days, adjust by 0.70 (257/365)
   3. $352.81 *0.70=$246.97
4. Apply adjustment for the percentage of total medical expenditures covered by CONY benefit plans
   1. 74.7% covered
   2. $246.97 * 0.747= $184.48
5. Calculate NYC program costs
   1. Costs are for employees only
   2. Cost for dependent= $0
6. Subtract NYC WW program costs from savings
   1. $184.48 - $0= $184.48

1. *October 17, 2016 letter from Milliman (Suzanne Taranto) to Mayor’s Office of Labor Relations (Claire Levitt)* [↑](#footnote-ref-1)
