# Supplementary material for: Financial implications of New York City’s weight management initiative
Source: PLoS One. 2021 Feb 11;16(2):e0246621. doi: 10.1371/journal.pone.0246621 (PMC7877753; doi:10.1371/journal.pone.0246621)
Supplement: S2 File — (RTF) [file pone.0246621.s003.rtf]

S2 File. STATA Code

*************************************************
***** WEIGHT WATCHERS PROJECT ****
*************************************************

** merging the two datafiles provided for 2019 data-pull

clear
set more off

**predict savings in medical expenditures based on BMI at enrolment, weight loss,
**duration of weight loss within the analysis period, and estimated age-specific probability of having diabetes

insheet using "Weight_data_2019_2.csv"

***********************
**** Cleaning Data ****
***********************
rename v* weight_week#, renumber(0) // rename the variables

drop week0 weight_week0
tostring week*, replace
save "weight_data_2019_2.dta", replace

clear
cd "C:\Users\CA39-1\Dropbox (Meteor)\rscrat_WeightWatchers\WW Manuscript Analysis Files 2020"
insheet using "Weight_data_2019_1.csv"
rename v* weight_week#, renumber(0) // rename the variables

drop week0 weight_week0
tostring week*, replace
append using weight_data_2019_2, force

drop if memberid==. 

save "weight_data_2019.dta", replace

******************************
***** DUPLICATES DO-FILE *****
******************************
clear
set more off

**predict savings in medical expenditures based on BMI at enrolment, weight loss,
**duration of weight loss within the analysis period

use "weight_data_2019.dta"

***********************
**** Cleaning Data ****
***********************

** convert string date variables to numeric version

forvalues i = 1/137 {
gen week_`i' = date(week`i', "MDY")
format week_`i' %td
drop week`i'
}

* preliminary elimination:
** remove duplicates from analysis
duplicates tag memberid, gen(isdup)
tab isdup

** keep only duplicates in the data set
drop if isdup==0

* gen enrollment date

gen enrollment_date = date(enrollmentdate, "MD20Y")
format enrollment_date %td
drop enrollmentdate

** getting age of participant
gen birth_date = date(birthdate, "MDY")
format birth_date %td
gen age = round((enrollment_date - birth_date)/365.25,1)

* gen subscription end date
gen subend_date = date(subscriptionenddate, "MDY")
format subend_date %td
drop subscriptionenddate

* gen b2b program end date

gen prog_end_date = date(b2bprogramenddate, "MDY")
format prog_end_date %td
drop b2bprogramenddate

** destring variables
encode sex, gen (gender) // gender==1 for female, ==2 for male
encode programtype, gen (program_type) // program_type==1 for monthly pass, ==2 for online
encode cancellation, gen (cancelled) //  ==1 for No, ==2 for Yes
encode populationtype, gen (pop_type)

** getting one row per member by formatting data from long to wide
sort memberid enrollment_date
bysort memberid (enrollment_date): gen dupcount=_n

rename weight_week* weight_week*_
rename week_* week_*_

drop sex programtype cancellation populationtype isdup monthlypassid uniqueid

reshape wide baselineheight birthdate baselineweight weight_week1_-weight_week137_ week_1_-week_137_ enrollment_date  birth_date subend_date prog_end_date age  gender  program_type  cancelled  pop_type , i(memberid) j(dupcount)

** check if reshaping worked
duplicates tag memberid, gen(dup)
tab dup //1,256 unique members after removing their duplicate recordings
drop dup
** save file
save "duplicates.dta", replace

**
order program_type*, after(baselineweight1)
egen program_type = rowlast(program_type1 - program_type5)
order cancelled*, after(baselineweight1)
egen cancelled = rowlast(cancelled1 - cancelled5)
order week_* weight_week* enrollment_date*, sequential after(pop_type5)

** switchers
gen switch=0
replace switch=1 if program_type1!= program_type2 
replace switch=1 if program_type2 != program_type3 & program_type3 !=.
replace switch=1 if program_type3 != program_type4 & program_type4 !=.
replace switch=1 if program_type4 != program_type5 & program_type5 !=.
tab switch

** 
rename baselineweight2 weight_week1_12
rename baselineweight3 weight_week1_13
rename baselineweight4 weight_week1_14
rename baselineweight5 weight_week1_15

rename enrollment_date2 week_1_12
rename enrollment_date3 week_1_13
rename enrollment_date4 week_1_14
rename enrollment_date5 week_1_15

order weight_week1_12 weight_week1_13 weight_week1_14 weight_week1_15, before(weight_week1_1)

local i = 1
foreach v of varlist weight_week1_12-weight_week137_5 {
   rename `v' weight`i'
   local i = `i' + 1
}

local i = 1
foreach v of varlist week_1_12-week_137_5 {
    rename `v' date`i'
	local i = `i' + 1
}

egen baselineweight = rowfirst(weight1-weight689) if baselineweight1==.

gen enrollment_date = 0
format enrollment_date %td
replace enrollment_date = date1 if baselineweight == weight1 & baselineweight1 ==.
replace enrollment_date = date2 if baselineweight == weight2 & baselineweight1 ==. & weight1==.
replace enrollment_date = date3 if baselineweight == weight3 & baselineweight1 ==. & weight1==. & weight2==.
replace enrollment_date = date4 if baselineweight == weight4 & baselineweight1 ==. & weight1==. & weight2==. & weight3==.
replace enrollment_date = date5 if baselineweight == weight5 & baselineweight1 ==. & weight1==. & weight2==. & weight3==. & weight4==.
replace enrollment_date = date6 if baselineweight == weight6 & baselineweight1 ==. & weight1==. & weight2==. & weight3==. & weight4==. & weight5==.

replace baselineweight = baselineweight1 if baselineweight1!=.
replace enrollment_date = enrollment_date1 if baselineweight1!=.

reshape long weight date, i(memberid) j(week)

** try to find as much similarity with the other main dataset
rename age1 age
rename gender1 gender
rename pop_type1 pop_type
rename baselineheight1 baselineheight
rename birth_date1 birth_date

save "duplicates_final.dta", replace

************************************
** WEIGHT LOSS COST SAVINGS MODEL **
************************************
clear
set more off

**predict savings in medical expenditures based on BMI at enrolment, weight loss,
**duration of weight loss within the analysis period

use "weight_data_2019.dta"

** convert string date variables to numeric version

forvalues i = 1/137 {
gen week_`i' = date(week`i', "MDY")
format week_`i' %td
drop week`i'
}

* preliminary elimination:
** remove duplicates from analysis
duplicates tag memberid, gen(isdup)
tab isdup 
drop if isdup!=0 
drop isdup

* gen enrollment date
gen enrollment_date = date(enrollmentdate, "MD20Y")
format enrollment_date %td
drop enrollmentdate

* gen subscription end date
gen subend_date = date(subscriptionenddate, "MDY")
format subend_date %td
drop subscriptionenddate

* gen b2b program end date

gen prog_end_date = date(b2bprogramenddate, "MDY")
format prog_end_date %td
drop b2bprogramenddate

** getting age of participant
gen birth_date = date(birthdate, "MDY")
format birth_date %td
gen age = round((enrollment_date - birth_date)/365.25,1)

** destring variables
encode sex, gen (gender) // gender==1 for female, ==2 for male
encode programtype, gen (program_type) // program_type==1 for monthly pass, ==2 for online
encode cancellation, gen (cancelled) //  ==1 for No, ==2 for Yes
encode populationtype, gen (pop_type) //1=Dependent, 2=domestic partner, 3=employee, 4=retiree, 5=spouse

****************************************
**** reshape data from wide to long ****
****************************************

local i = 1
foreach v of varlist weight_week1-weight_week137 {
   rename `v' weight`i'
   local i = `i' + 1
}

local i = 1
foreach v of varlist week_1-week_137 {
    rename `v' date`i'
	local i = `i' + 1
}

reshape long weight date, i(memberid) j(week)

append using duplicates_final

save "weight_data_2019_long.dta", replace

clear 
use "weight_data_2019_long.dta"

** ignore Subscription end date
drop subend_date

* Retrieve last assessment date within POA
bysort memberid: egen temp = max(date) if date >= td(01jun2016) & date < td(01jun2017)
bysort memberid: egen lastdatePOA = min(temp)
drop temp
format lastdatePOA %td

* Retrieve first assessment date past POA
bysort memberid: egen temp = min(date) if date > td(31may2017) & date < td(01sep2017)
bysort memberid: egen firstdatePost = min(temp)
drop temp
format firstdatePost %td

* Retrieve last assessment date prior to POA
bysort memberid: egen temp = max(date) if date < td(01jun2016) 
bysort memberid: egen lastdatePre = min(temp)
drop temp
format lastdatePre %td

* Retrieve first assessment date within POA
bysort memberid: egen temp = min(date) if date >= td(01jun2016) & date < td(01jun2017)
bysort memberid: egen firstdatePOA = min(temp)
drop temp
format firstdatePOA %td

* does person have a weight prior to evaluation period
bysort memberid: egen temp = count(date) if date < td(01jun2016)
bysort memberid: egen nbdatespre = min(temp) 
replace nbdatespre = 0 if nbdatespre == .
drop temp

* does person have a weight during evaluation period
bysort memberid: egen temp = count(date) if date >= td(01jun2016) & date < td(01jun2017)
bysort memberid: egen nbdatesPOA = min(temp)
replace nbdatesPOA = 0 if nbdatesPOA == .
drop temp

* does person have a weight during grace period
bysort memberid: egen temp = count(date) if date > td(31may2017) & date < td(01sep2017)
bysort memberid: egen nbdatespostPOA = min(temp)
replace nbdatespostPOA = 0 if nbdatespostPOA == .
drop temp

* scenario variable 
gen scenario=.
bysort memberid: replace scenario=1 if baselineweight==. & nbdatespre==0 & nbdatesPOA==0 & nbdatespostPOA==0 
bysort memberid: replace scenario=2 if enrollment_date < td(1jun2016) & nbdatespre==0 & nbdatesPOA==0 & nbdatespostPOA==0
bysort memberid: replace scenario=3 if enrollment_date >= td(1jun2016) & enrollment_date < td(1jun2017) & nbdatespre==0 & nbdatesPOA==0 & nbdatespostPOA==0
bysort memberid: replace scenario=4 if enrollment_date > td(31may2017) & enrollment_date < td(01sep2017) & nbdatespre==0 & nbdatesPOA==0 & nbdatespostPOA==0
bysort memberid: replace scenario=5 if enrollment_date < td(1jun2016) & nbdatespre==1 & nbdatesPOA==0 & nbdatespostPOA==0
bysort memberid: replace scenario=6 if enrollment_date < td(1jun2016) & nbdatespre==0 & nbdatesPOA==1 & nbdatespostPOA==0
bysort memberid: replace scenario=7 if enrollment_date < td(1jun2016) & nbdatespre==0 & nbdatesPOA==0 & nbdatespostPOA==1
bysort memberid: replace scenario=8 if enrollment_date >= td(1jun2016) & enrollment_date < td(1jun2017) & nbdatespre==0 & nbdatesPOA==0 & nbdatespostPOA==1
bysort memberid: replace scenario=9 if enrollment_date >= td(1jun2016) & enrollment_date < td(1jun2017) & nbdatespre==0 & nbdatesPOA==1 & nbdatespostPOA==0
bysort memberid: replace scenario=10 if enrollment_date > td(31may2017) & enrollment_date < td(01sep2017) & nbdatespre==0 & nbdatesPOA==0 & nbdatespostPOA==1
bysort memberid: replace scenario=11 if enrollment_date < td(1jun2016) & nbdatespre>=2 & nbdatesPOA==0 & nbdatespostPOA==0
bysort memberid: replace scenario=12 if enrollment_date < td(1jun2016) & nbdatespre==0 & nbdatesPOA>=2 & nbdatespostPOA==0
bysort memberid: replace scenario=13 if enrollment_date < td(1jun2016) & nbdatespre==0 & nbdatesPOA>=1 & nbdatespostPOA>=1
bysort memberid: replace scenario=14 if enrollment_date < td(1jun2016) & nbdatespre==0 & nbdatesPOA==0 & nbdatespostPOA>=2
bysort memberid: replace scenario=15 if enrollment_date >= td(1jun2016) & enrollment_date < td(1jun2017) & nbdatespre==0 & nbdatesPOA==0 & nbdatespostPOA>=2
bysort memberid: replace scenario=16 if enrollment_date >= td(1jun2016) & enrollment_date < td(1jun2017) & nbdatespre==0 & nbdatesPOA>=2 & nbdatespostPOA==0
bysort memberid: replace scenario=17 if enrollment_date >= td(1jun2016) & enrollment_date < td(1jun2017) & nbdatespre==0 & nbdatesPOA>=1 & nbdatespostPOA>=1
bysort memberid: replace scenario=18 if enrollment_date < td(1jun2016) & nbdatespre>=1 & nbdatesPOA>=1 & nbdatespostPOA==0
bysort memberid: replace scenario=19 if enrollment_date < td(1jun2016) & nbdatespre>=1 & nbdatesPOA==0 & nbdatespostPOA>=1
bysort memberid: replace scenario=20 if enrollment_date > td(31may2017) & enrollment_date < td(01sep2017) & nbdatespre==0 & nbdatesPOA==0 & nbdatespostPOA>=2
replace scenario=0 if scenario==.

preserve
collapse (max) scenario, by (memberid)
tab scenario  //19,371 unique members
restore

* duration 
gen POA_start_date=mdy(6,1,2016)
gen POA_end_date=mdy(5,31,2017)
gen grace_period_end_date=mdy(8,31,2017)
format POA_end_date POA_start_date grace_period_end_date %td

** require first date and last date for savings
gen first_date=0
replace first_date=enrollment_date if scenario==2 | scenario==3 | scenario==4 | scenario==8 | scenario==9 | scenario==15 | scenario==16 | scenario==17
replace first_date=POA_start_date if scenario==5 | scenario==6 | scenario==7 | scenario==11 | scenario==12 | scenario==13 | scenario==14 | scenario==18 | scenario==19
replace first_date=. if scenario==0

gen last_date=0
bysort memberid: replace last_date=. if scenario==2 | scenario==3 | scenario==4 | scenario==0
bysort memberid: replace last_date=POA_start_date if scenario==5 | scenario==11 
bysort memberid: replace last_date=lastdatePOA if scenario==6 | scenario==9 | scenario==12 | scenario==16 | scenario==18
bysort memberid: replace last_date=POA_end_date if scenario==7 | scenario==8 | scenario==13 | scenario==14 | scenario==15 | scenario==17 | scenario==19

format first_date last_date %td

bysort memberid: gen duration = last_date - first_date

* weight change

bysort memberid: gen temp=0
bysort memberid: replace temp=weight if lastdatePOA==date
bysort memberid: egen weightPOA=max(temp)
drop temp

bysort memberid: gen temp=0
bysort memberid: replace temp=weight if firstdatePost==date
bysort memberid: egen weightPost=max(temp)
replace weightPost=. if firstdatePost==.
drop temp

bysort memberid: gen temp=0
bysort memberid: replace temp=weight if lastdatePre==date
bysort memberid: egen weightPre=max(temp)
drop temp

bysort memberid: gen temp=0
bysort memberid: replace temp=weight if firstdatePOA==date
bysort memberid: egen firstweightPOA=max(temp)
drop temp

** weight adjustment for first_weight (pre POA and POA)
gen A1 = firstdatePOA - lastdatePre
gen B1 = POA_start_date - lastdatePre
gen C1 = firstdatePOA - POA_start_date

gen W11 = B1/A1
gen W21 = C1/A1

bysort memberid: gen first_weight_adj = W11*firstweightPOA + W21*weightPre 

** weight adjustment for first_weight (enrollment weight and grace period)
gen A2 = firstdatePost - enrollment_date if enrollment_date < POA_start_date
gen B2 = POA_start_date - enrollment_date if enrollment_date < POA_start_date
gen C2 = firstdatePost - POA_start_date

gen W12 = B2/A2
gen W22 = C2/A2

bysort memberid: gen first_weight_adj_grace = W12*weightPost + W22*baselineweight if enrollment_date < POA_start_date 

** weight adjustment for first_weight (enrollment weight and POA)
gen A3 = firstdatePOA - enrollment_date if enrollment_date < POA_start_date
gen B3 = POA_start_date - enrollment_date if enrollment_date < POA_start_date
gen C3 = firstdatePOA - POA_start_date

gen W13 = B3/A3
gen W23 = C3/A3

bysort memberid: gen first_weight_adj_enroll = W13*firstweightPOA + W23*baselineweight if enrollment_date < POA_start_date 

** weight adjustment for first_weight (pre POA and grace period)
gen A4 = firstdatePost - lastdatePre 
gen B4 = POA_start_date - lastdatePre 
gen C4 = firstdatePost - POA_start_date

gen W14 = B4/A4
gen W24 = C4/A4

bysort memberid: gen first_weight_adj_grace_pre = W14*weightPost + W24*weightPre 

** weight adjustment for last_weight (POA and grace period)
gen A = firstdatePost - lastdatePOA  // e.g. difference between April 15 and June 5
gen B = POA_end_date - lastdatePOA // e.g. difference between April 15 and May 31
gen C = firstdatePost - POA_end_date // e.g. difference between May 31 and June 5

gen W1 = B/A
gen W2 = C/A

bysort memberid: gen last_weight_adj = W1*weightPost + W2*weightPOA

** weight adjustment for last_weight (enrollment weight pre POA and grace period)

gen A5 = firstdatePost - enrollment_date if enrollment_date < POA_start_date
gen B5 = POA_end_date - enrollment_date if enrollment_date < POA_start_date
gen C5 = firstdatePost - POA_end_date

gen W15 = B5/A5
gen W25 = C5/A5

bysort memberid: gen last_weight_enroll_grace = W15*weightPost + W25*baselineweight if enrollment_date < POA_start_date

** weight adjustment for last_weight (enrollment weight POA and grace period)

gen A6 = firstdatePost - enrollment_date if enrollment_date >= POA_start_date & enrollment_date <= POA_end_date
gen B6 = POA_end_date - enrollment_date if enrollment_date >= POA_start_date & enrollment_date <= POA_end_date
gen C6 = firstdatePost - POA_end_date

gen W16 = B6/A6
gen W26 = C6/A6

bysort memberid: gen last_weight_POAenroll_grace = W16*weightPost + W26*baselineweight if enrollment_date >= POA_start_date & enrollment_date <= POA_end_date

** weight adjustment for last_weight (pre POA and grace period)

gen A7 = firstdatePost - lastdatePre 
gen B7 = POA_end_date - lastdatePre 
gen C7 = firstdatePost - POA_end_date

gen W17 = B7/A7
gen W27 = C7/A7

bysort memberid: gen last_weight_pre_grace = W17*weightPost + W27*weightPre 

** first and last weights
gen first_weight=0
bysort memberid: replace first_weight=baselineweight if scenario==2 | scenario==3 | scenario==4 | scenario==8 | scenario==9 | scenario==15 | scenario==16 | scenario==17
bysort memberid: replace first_weight=first_weight_adj if scenario==18 
bysort memberid: replace first_weight=first_weight_adj_grace if scenario==7 | scenario==14
bysort memberid: replace first_weight=first_weight_adj_enroll if scenario==6 | scenario==12 | scenario==13  
bysort memberid: replace first_weight=first_weight_adj_grace_pre if scenario==19 
bysort memberid: replace first_weight=. if scenario==5 | scenario==11

gen last_weight=0
bysort memberid: replace last_weight=. if scenario==2 | scenario==3 | scenario==4 | scenario==5 | scenario==11
bysort memberid: replace last_weight=weightPOA if scenario==6 | scenario==9 | scenario==12 | scenario==16 |scenario==18
bysort memberid: replace last_weight=last_weight_adj if scenario==13 | scenario==17 
bysort memberid: replace last_weight=last_weight_enroll_grace if scenario==7 | scenario==14
bysort memberid: replace last_weight=last_weight_POAenroll_grace if scenario==8 | scenario==15
bysort memberid: replace last_weight=last_weight_pre_grace if scenario==19

** weight change
bysort memberid: gen weight_change = first_weight - last_weight

bysort memberid: gen BMI_reduction = (weight_change/baselineweight)*100
bysort memberid: replace BMI_reduction=round(BMI_reduction,1) if BMI_reduction!=2.5

bysort memberid: gen BMI_enrollment_original = ((baselineweight/(baselineheight*baselineheight))*703)
bysort memberid: replace BMI_enrollment_original = . if baselineheight==. | baselineweight==.
bysort memberid: replace BMI_enrollment_original = round(BMI_enrollment_original,1)

** Treat starting BMI above 45 as 45

gen BMI_enrollment = BMI_enrollment_original

preserve
collapse (max) BMI_enrollment_original, by(memberid)
count if BMI_enrollment_original>45 & BMI_enrollment_original!=. 
tab BMI_enrollment_original
count if BMI_enrollment_original==.
restore

replace BMI_enrollment = 45 if BMI_enrollment_original>45 & BMI_enrollment_original!=. 

** incorporting Cawley et al Tables into the data
merge m:1 BMI_enrollment using "cawley_table1_inflated_$2017.dta"
drop _merge 

// return savings associated with the age, BMI reduction and starting BMI

gen saving_red_BMI=0
forvalues x = 1/25 {
  bysort BMI_enrollment: replace saving_red_BMI = BMI_red_`x' if BMI_reduction==`x'
  bysort BMI_enrollment: replace saving_red_BMI = -BMI_red_`x' if BMI_reduction==-`x'
}
bysort BMI_enrollment: replace saving_red_BMI = BMI_red_2_5 if BMI_reduction==2.5
bysort BMI_enrollment: replace saving_red_BMI = -BMI_red_2_5 if BMI_reduction==-2.5

bysort memberid: gen savings_1 = saving_red_BMI

* adjustments to savings based on duration and CONY benefits plan
bysort memberid: gen duration_month = (duration)* (12/365)
bysort memberid: gen BMI_reduction_month = (BMI_reduction)/duration_month

bysort memberid: gen eval_period = (duration/365)
bysort memberid: gen savings_2 = savings_1*eval_period
bysort memberid: gen savings_final = savings_2*0.747 // adjustment

bysort memberid: replace savings_final=0 if BMI_enrollment_original<30
bysort memberid: replace savings_final=0 if BMI_reduction<2.5 & BMI_reduction>0
bysort memberid: replace savings_final=0 if BMI_reduction>-2.5 & BMI_reduction<0
bysort memberid: replace savings_final=0 if duration<90 & duration!=0
bysort memberid: replace savings_final=0 if BMI_reduction==0 
bysort memberid: replace savings_final=0 if duration==0

** assign costs
//monthly cost = $7 if online, $15 if monthly-pass; only employees generate costs

bysort memberid: gen monthly_cost = 0
bysort memberid: replace monthly_cost=7 if program_type==2 & pop_type==3
bysort memberid: replace monthly_cost=15 if program_type==1 & pop_type==3

** cap change in BMI to 20 for values between 20 and 25

preserve
collapse (max) BMI_reduction, by (memberid)
count if BMI_reduction>20 & BMI_reduction<=25 
count if BMI_reduction<-20 & BMI_reduction>=-25
restore
bysort memberid: replace BMI_reduction=20 if BMI_reduction>20 & BMI_reduction<=25
bysort memberid: replace BMI_reduction=-20 if BMI_reduction<-20 & BMI_reduction>=-25

save "weight_data_long_clean_$2017inflation_20c1.dta", replace 

**********************
**** Out of Range ****
**********************

clear
use "weight_data_long_clean_$2017inflation_20c1.dta"

gen employees=1
replace employees=0 if pop_type!=3

* drop if age out of range

gen age_invalid=0
replace age_invalid=1 if age<18 | age>64
preserve
collapse (max) age baselineweight baselineheight BMI_enrollment_original BMI_reduction BMI_reduction_month duration duration_month age_invalid employees, by (memberid)
count if age<18
count if age>64
count if age>=18 & age<20
bysort employees: count if age_invalid==1
restore
drop if age_invalid==1

* drop if BMI out of range
preserve
collapse (max) BMI_enrollment_original baselineweight baselineheight, by (memberid)
count if BMI_enrollment_original<26  
count if BMI_enrollment_original>45 & BMI_enrollment_original!=. 
count if BMI_enrollment_original==. 
tab BMI_enrollment_original, missing
restore
drop if BMI_enrollment_original<26  

* drop if enrollment_date > 31may2017
preserve
collapse (max) enrollment_date scenario, by(memberid)
count if enrollment_date > td(31may2017)
restore
drop if enrollment_date > td(31may2017) 

* drop if enrolled less than 2 months ago

preserve
collapse (max) duration_month duration enrollment_date, by (memberid)
count if enrollment_date > td(31mar2017) & duration!=0 & duration < 60 
restore
drop if enrollment_date > td(31mar2017) & duration!=0 & duration < 60 

* 
preserve
collapse (max) scenario employees, by(memberid)
tab scenario
tab employees
restore

preserve
collapse (max) scenario nbdatespostPOA, by (memberid)
tab scenario if nbdatespostPOA>0 
restore

*******************************
** out of range for savings
******************************

gen out_of_range=0
** drop if baseline weight is missing
preserve
collapse (max) baselineweight, by(memberid)
count if baselineweight==.
restore 
replace out_of_range=1 if baselineweight==.

** drop if baseline height is missing
preserve
collapse (max) baselineheight, by(memberid)
count if baselineheight==.
restore 
replace out_of_range=1 if baselineheight==.

* drop if one weight only:
count if scenario==2 | scenario==3 | scenario==4
bysort memberid: gen one_weight=0
bysort memberid: replace one_weight=1 if scenario==2 | scenario==3 | scenario==4
preserve
collapse (max) one_weight scenario duration, by(memberid)
count if one_weight==1 
restore
replace out_of_range=1 if one_weight==1 

*baseline height and weight

preserve
collapse (max) baselineweight, by (memberid)
count if baselineweight>700 
count if baselineweight<104
restore

replace out_of_range=1 if baselineweight>700 | baselineweight<104

preserve
collapse (max) baselineheight, by (memberid)
count if baselineheight>84 
count if baselineheight<58
restore

replace out_of_range=1 if baselineheight>84 | baselineheight<58

preserve
collapse (max) duration duration_month BMI_reduction BMI_reduction_month, by(memberid)
count if BMI_reduction>25
restore

replace out_of_range=1 if BMI_reduction>25 

preserve
collapse (max) duration duration_month BMI_reduction BMI_reduction_month, by(memberid)
count if BMI_reduction<-25
restore

replace out_of_range=1 if BMI_reduction<-25 

preserve
collapse (max) duration duration_month BMI_reduction BMI_reduction_month, by(memberid)
count if BMI_reduction_month>8.5 & duration!=0
count if BMI_reduction_month<-8.5 & duration!=0
restore

replace out_of_range=1 if BMI_reduction_month>8.5 & duration!=0
replace out_of_range=1 if BMI_reduction_month<-8.5 & duration!=0

preserve
collapse (max) out_of_range duration employees, by(memberid)
tab out_of_range
tab employees if out_of_range==0
count if out_of_range==1
restore

***************
** save file **
***************
encode groups, gen (enroll_BMI_cat)

save "weight_data_long_clean_2019_$2017inflation_20c1.dta", replace

** duration for costs:
codebook prog_end_date

gen last_date_cost = prog_end_date
replace last_date_cost = POA_end_date if prog_end_date==.
replace last_date_cost = POA_end_date if prog_end_date>POA_end_date
format last_date_cost %td

bysort memberid: gen duration_cost = last_date_cost - first_date
bysort memberid: gen duration_month_cost = (duration_cost)* (12/365)

** collapse the data to member level
** variables required: savings_final monthly_cost duration duration_month age gender program_type cancelled pop_type weight_change BMI_enrollment prob_diabetes BMI_reduction BMI_reduction_month out_of_range 

collapse (max) one_weight baselineheight baselineweight age_invalid prog_end_date enrollment_date POA_start_date POA_end_date grace_period_end_date first_date last_date last_date_cost enroll_BMI_cat savings_final monthly_cost duration duration_month age gender program_type cancelled pop_type weight_change first_weight last_weight BMI_enrollment BMI_enrollment_original BMI_reduction BMI_reduction_month scenario out_of_range duration_cost duration_month_cost employees, by (memberid)

save "weight_data_collapsed_2019_$2017inflation_20c1.dta", replace

*****************************
***** results generator *****
*****************************

clear
set more off
use "weight_data_collapsed_2019_$2017inflation_FIN.dta"

**
codebook duration_month
replace duration_month=0 if duration_month==.
codebook duration_month

** duration rounded upward to full month for cost calculation
gen duration_for_cost = duration_month_cost
replace duration_for_cost = ceil(duration_month_cost)
**
** clean up savings
replace savings_final=0 if savings_final==. & scenario==3
**
gen total_cost = monthly_cost*duration_for_cost 
replace total_cost = monthly_cost if duration_month_cost==0
egen total_savings = total(savings_final) if out_of_range==0 
egen total_costs = total(total_cost) 

gen net_saving = savings_final - total_cost

** to get S.Ds for per capita figures:

bysort employees program_type: sum savings_final, detail
bysort employees: sum savings_final, detail
bysort employees: sum net_saving, detail
bysort employees program_type: sum total_cost, detail
bysort employees: sum total_cost, detail

****************************
**** SUMMARY STATISTICS ****
****************************
** program_type==1 for monthly pass, ==2 for online // gender==1 for female, ==2 for male // cancelled==1 for No,==2 for Yes //
//1=Dependent, 2=domestic partner, 3=employee, 4=retiree, 5=spouse

bysort memberid: gen weight_loss_per_month = (weight_change/duration_month)

local list_vars "age BMI_enrollment duration_month weight_change BMI_reduction BMI_reduction_month weight_loss_per_month duration_for_cost"

foreach var in `list_vars' {
  sum `var' 
}

local list_var1 "gender program_type cancelled pop_type"

foreach var in `list_var1' {
  tab `var' 
}

local list_vars "age BMI_enrollment duration_month weight_change BMI_reduction BMI_reduction_month weight_loss_per_month duration_for_cost"

foreach var in `list_vars' {
bysort employees:  sum `var' 
}

local list_var1 "gender program_type cancelled pop_type"

foreach var in `list_var1' {
bysort employees:  tab `var' 
}

local list_vars "age BMI_enrollment duration_month weight_change BMI_reduction BMI_reduction_month weight_loss_per_month"

foreach var in `list_vars' {
sum `var' if out_of_range==0 & employees==1
}

local list_var1 "gender cancelled pop_type"

foreach var in `list_var1' {
bysort program_type:  tab `var' 
}

bysort program_type:  sum weight_change
bysort employees: sum weight_change
bysort employees program_type: sum weight_change
bysort employees program_type: sum weight_change, detail
bysort employees : sum duration_month, detail
bysort employees : sum duration_for_cost, detail

*********************************
**** SAVINGS BY PROGRAM TYPE ****
*********************************

bysort employees: tab program_type 

bysort program_type employees: egen savings_cat_prog = total(savings_final) if out_of_range==0 
bysort employees: tab savings_cat_prog program_type, missing 

bysort program_type employees: egen cost_cat_prog = total(total_cost) 
bysort employees: tab cost_cat_prog program_type, missing 

***********************************
**** SAVINGS BY ENROLLMENT BMI ****
***********************************
** BMI_cat" 1=obese I, 2= Obese II, 3= Obese III, 4=Overweight
bysort employees: tab enroll_BMI_cat, missing 

bysort enroll_BMI_cat employees: egen savings_cat_enr_BMI = total(savings_final) if out_of_range==0 
bysort employees: tab savings_cat_enr_BMI enroll_BMI_cat, missing 

bysort enroll_BMI_cat employees: egen cost_cat_enr_BMI = total(total_cost) 
bysort employees: tab cost_cat_enr_BMI enroll_BMI_cat, missing 

**********************************
**** SAVINGS BY BMI REDUCTION ****
**********************************

gen BMI_red = BMI_reduction
replace BMI_red = round(BMI_reduction, 0.5)

gen BMI_red_cat=0
replace BMI_red_cat=1 if BMI_red>=0.01 & BMI_red<2.5
replace BMI_red_cat=2 if BMI_red>=2.5 & BMI_red<5
replace BMI_red_cat=3 if BMI_red>=5 & BMI_red<7.5
replace BMI_red_cat=4 if BMI_red>=7.5 & BMI_red<10
replace BMI_red_cat=5 if BMI_red>=10 & BMI_red!=.
replace BMI_red_cat=6 if BMI_red==0
replace BMI_red_cat=7 if BMI_red<0

bysort employees: tab BMI_red_cat, missing 

bysort BMI_red_cat employees: egen savings_cat_BMI_red = total(savings_final) if out_of_range==0 
bysort employees: tab savings_cat_BMI_red BMI_red_cat, missing 

bysort BMI_red_cat employees: egen cost_cat_BMI_red = total(total_cost) 
bysort employees: tab cost_cat_BMI_red BMI_red_cat, missing 

**************************
***** END OF DO-FILE *****
**************************
