# Supplementary material for: Financial implications of New York City’s weight management initiative
Source: PLoS One. 2021 Feb 11;16(2):e0246621. doi: 10.1371/journal.pone.0246621 (PMC7877753; doi:10.1371/journal.pone.0246621)
Supplement: Attachment — Submitted filename: Comments to the authors.pdf [file pone.0246621.s004.pdf]

Comments to the Author

This paper describes simulated medical cost savings due to weight loss of participants in a Weight Watchers program. The paper is motivated by trying to establish cost savings to employers by subsidizing membership fees to the Weight Watchers program. The paper is in general well written and clear to follow. That being said the analysis is based on some broad assumptions and there are some key missing features which it would be helpful to include.

Introduction –

The authors use the values of $260 on the low end and $12,548 on the high end of medical expenditure savings for weight loss taken from Crowley et al. The authors do not mention the high standard errors with these measurements or the overall analysis of the MEPS data that is conducted by Crowley et al showing significantly lower savings. Both should be addressed.

If the state is not subsidizing dependent enrollment in WW what is the states roll in providing this service to dependents?

The authors claim on line 50-52 that WW produces weight loss of approximately 5% and is cost effective. Is this an average or estimated weight loss percentage? Additionally, the reference used for the claim of cost effectiveness (29) is based on a study of prediabetic subjects not the general population. This should be addressed by the authors.

The term disease burden on line 45 is not well defined and should be explained.

Line 54 The authors state that the goal of the study is to quantify the total and per capita net annual medical expenditure savings. This appears inconsistent with the Authors' Assumption #9.

Sample

Is the 430,000 the number of employees that meet the study's criteria or all NYC employees?

The authors do not state why the cutoff BMI for eligibility is 25 kg/m^2 or if participants with a starting BMI between 25-26 are included in cost savings or city cost calculations.

Do other dependents include children? Are medical expenditure savings the same for all age ranges?

Are there age cutoffs for the study? In Crowley et all, the estimates are for a population of adults between the ages of 24 and 64 years with biological children between the ages of 11 years (132 months) and 20 years (240 months). How does this compare to the sample in this study?

Intervention

What is behavioral skill?

Measures

Are the authors extrapolating partial year savings to full annual savings? This is not clear.

Data

The authors do not explain how they impute diabetes prevalence.

Analysis

Line 121 - The authors state that the if a participant did not have a disenrollment date they are assumed to be enrolled till the end of the evaluation period. Are there weight measurements for all these subjects at the end of the evaluation period to demonstrate that the weight loss was maintained during this time? Otherwise, what indications do the authors have that indicate weight loss besides the participants continuing to pay for their portion of program cost.

The paragraph starting on line 125 is confusing. If the evaluation period is between June 2016 and May 31, 2017 why are there weight measurements from July and August 2017? How many subjects did this occur with and were they still enrolled in the WW program at the subsidized rate from the end of May until their final weight measurements? If not, how do the authors attribute any weight loss during this time to the WW program?

Line 131 – For participants with a duration of zero, are the costs incurred by the city for these participants included in the cost savings calculation?

Line 135 – The authors state that if participants have an additional weight recorded in the three months after the evaluation period their final weight is a weighted average of the final two weights. How is this weighted average calculated and how many participants did this include? Were these participants still enrolled in the program at the subsidized rate from the end of May until their final weight measurements, if not how is weight loss after the end of the evaluation period attributed to the weight watchers' program.

Assumptions - While briefly discussed, the assumptions the authors make to extrapolate outside published literature on cost saving due to weight loss are very broad. These assumptions should be addressed up front in the paper as they cast doubt on the validity of the cost savings analysis.

Assumption 1 - Extrapolating for cost saving for under 5% weight loss especially at lower starting BMI does not appear to be supported by reference 9 and 10.

Assumption 2 – Extrapolating cost savings for subjects under a starting BMI of 30, especial especially for weight loss of under 5 percent is a large assumption that should be addressed up front. A 5'7" male with a weight of 170lbs has a BMI of 26.62. A 2.5% reduction in weight is only 4.25lbs which is within established values for normal daily weight fluctuation.

Cost savings calculations should be reported without extrapolating below a starting BMI of 30 and without extrapolating below 5% weight loss for comparison.

Assumption 3 – The author gives no explanation for this recoding of the data.

Assumption 4 – The authors state that only participants with more than one weight recorded were eligible to contribute to savings. Did these individuals contribute to the city's cost for subsidizing the program?

Assumption 8 – The authors appear to treat all medical expenditures as equal regarding cost. Do the authors take into account different costs for covered vs not covered procedures?

Assumption 9 – The authors assume that weight loss is immediate for all participants which is an unreasonable assumption. Additionally, the authors assume that weight loss is maintained after an individual's final weight in. Is there any evidence that this is actually the case?

Sample Characteristics

The authors state that 91% of employees and 78% of dependents were female. Are there any differences between the cost savings due to weight loss for males vs females?

Duration

The authors state that 47.8% of employees and 45.1% of dependents did not generate cost savings. Did these participants contribute to the cost NYC incurred in the cost savings calculations?

Limitations

The authors state that self-reported weights for digital participants (42% of overall sample) were similar to other online programs that relied on measured weight. What programs are the authors using for comparison and what criteria is used to determine similarity of measurements?

The authors do not acknowledge or discuss possible increase in medical expenditures due to weight loss. i.e. An individual that needs a hip replacement but can not get the surgery until loosing weight. An individual in this situation would incur increased medical expenditures due to weight loss rather than cost savings. This should be discussed as a limitation of this analysis.
