# Supplementary material for: Financial implications of New York City’s weight management initiative
Source: PLoS One. 2021 Feb 11;16(2):e0246621. doi: 10.1371/journal.pone.0246621 (PMC7877753; doi:10.1371/journal.pone.0246621)
Supplement: Attachment — Submitted filename: Response to Reviewers.docx [file pone.0246621.s005.docx]

Journal Requirements:

1. When submitting your revision, we need you to address these additional requirements.
   Please ensure that your manuscript meets PLOS ONE's style requirements, including those for file naming. The PLOS ONE style templates can be found at
   <http://www.journals.plos.org/plosone/s/file?id=wjVg/PLOSOne_formatting_sample_main_body.pdf> and <http://www.journals.plos.org/plosone/s/file?id=ba62/PLOSOne_formatting_sample_title_authors_affiliations.pdf>

We have addressed this.

2. Please ensure that you include a title page within your main document. We do appreciate that you have a title page document uploaded as a separate file, however, as per our author guidelines (<http://journals.plos.org/plosone/s/submission-guidelines#loc-title-page>) we do require this to be part of the manuscript file itself and not uploaded separately.

We have addressed this.

3. Could you therefore please include the title page into the beginning of your manuscript file itself, listing all authors and affiliations.

We have addressed this.

4. We note that you have indicated that data from this study are available upon request. PLOS only allows data to be available upon request if there are legal or ethical restrictions on sharing data publicly. For information on unacceptable data access restrictions, please see <http://journals.plos.org/plosone/s/data-availability#loc-unacceptable-data-access-restrictions>.
   In your revised cover letter, please address the following prompts:
   a) If there are ethical or legal restrictions on sharing a de-identified data set, please explain them in detail (e.g., data contain potentially identifying or sensitive patient information) and who has imposed them (e.g., an ethics committee). Please also provide contact information for a data access committee, ethics committee, or other institutional body to which data requests may be sent.
   b) If there are no restrictions, please upload the minimal anonymized data set necessary to replicate your study findings as either Supporting Information files or to a stable, public repository and provide us with the relevant URLs, DOIs, or accession numbers. Please see <http://www.bmj.com/content/340/bmj.c181.long> for guidelines on how to de-identify and prepare clinical data for publication. For a list of acceptable repositories, please see <http://journals.plos.org/plosone/s/data-availability#loc-recommended-repositories>.
   We will update your Data Availability statement on your behalf to reflect the information you provide.

Due to the contractual requirements by the City of New York we are unable to share this data without a data use agreement in place. If a requestor wishes to replicate this work, they can send a data request to Eric Finkelstein at [eric.finkelstein@duke-nus.edu.sg](mailto:eric.finkelstein@duke-nus.edu.sg) who will then initiate the process of executing the agreement. Upon approval, de-identified data will be made available to the requestor for the approved analyses.

5. Thank you for stating the following in the Financial Disclosure section: This work was supported by WW International. We note that one or more of the authors are employed by a commercial company: WW International. Please provide an amended Funding Statement declaring this commercial affiliation, as well as a statement regarding the Role of Funders in your study. If your commercial affiliation did play a role in your study, please state and explain this role within your updated Funding Statement.

Financial Disclosures: As noted in the original submission, this work was funded by WW International, a global wellness company. We now also note the following: *All authors, including two from the funder, played a role in the conception and design of this study. Agrawal conducted all data analyses with input and oversight from Finkelstein. These analyses were conducted independent of the funder. Finkelstein and Agrawal led manuscript writing with input from all authors. Publication of results was agreed at the time of execution of the contract and independent of what the final results showed.*

6. Please also provide an updated Competing Interests Statement declaring this commercial affiliation along with any other relevant declarations relating to employment, consultancy, patents, products in development, or marketed products, etc.  Within your Competing Interests Statement, please confirm that this commercial affiliation does not alter your adherence to all PLOS ONE policies on sharing data and materials by including the following statement: "This does not alter our adherence to  PLOS ONE policies on sharing data and materials.” (as detailed online in our guide for authors <http://journals.plos.org/plosone/s/competing-interests>) . If this adherence statement is not accurate and  there are restrictions on sharing of data and/or materials, please state these. Please note that we cannot proceed with consideration of your article until this information has been declared. Please know it is PLOS ONE policy for corresponding authors to declare, on behalf of all authors, all potential competing interests for the purposes of transparency. PLOS defines a competing interest as anything that interferes with, or could reasonably be perceived as interfering with, the full and objective presentation, peer review, editorial decision-making, or publication of research or non-research articles submitted to one of the journals. Competing interests can be financial or non-financial, professional, or personal. Competing interests can arise in relationship to an organization or another person. Please follow this link to our website for more details on competing interests: <http://journals.plos.org/plosone/s/competing-interests>

We have now added the following:

*Competing Interests: Authors Wojtanowski, Tringali, and Foster are employees of WW International. Finkelstein is a paid consultant and paid member of the WW International Scientific Advisory Board. These competing interests do not alter our adherence to PLOS ONE policies on sharing data and materials.*

7. PLOS requires an ORCID iD for the corresponding author in Editorial Manager on papers submitted after December 6th, 2016. Please ensure that you have an ORCID iD and that it is validated in Editorial Manager. To do this, go to ‘Update my Information’ (in the upper left-hand corner of the main menu), and click on the Fetch/Validate link next to the ORCID field. This will take you to the ORCID site and allow you to create a new iD or authenticate a pre-existing iD in Editorial Manager. Please see the following video for instructions on linking an ORCID iD to your Editorial Manager account: <https://www.youtube.com/watch?v=_xcclfuvtxQ>

This has been done as requested.

Additional Editor Comments:

The reviewers have noted concerns with the assumptions, the statistical reporting and the conclusions derived from these. The editor suggests that the results may not be extraordinary if the assumptions are revisited, however, even if they are not, reporting the results are interesting. Toning down the claims of the program as suggested by the reviewers is critical.

*We have addressed all reviewers as noted below, including toning down the claims of the program.*

**Reviewers' comments:
Reviewer #1:**

1) This is a well written paper to quantify the annual medical expenditure savings from an employee subsidized WW workshop or digital program weight management intervention. Overall, I believe this is a well conducted study with limitations noted in the discussion and abstract. Needed revisions are listed below.

*We appreciate the positive comments from the reviewer. Specific responses to comments are noted below.*

Abstract:
2) Important to highlight that only 51% of employee and 54.1% of dependent enrollees were eligible to generate savings

*We now note in the abstract: Based on the data and assumptions, 51% of employees and 54% of dependents were eligible to contribute to savings.’*

3) If space allows, would also be good to highlight that the majority of savings (73% for both employees and dependents) came from enrollees with class II or III obesity.

*We now note: Over 80% of savings came from participants in the Obese III category.*

Methods:
4) Line 64. Note that height was also collected.

*We now note in the Data Section: Participant-level data from the WW database (self-reported date of birth* ***and height at enrollment****)…. was used to conduct the analysis.*

5) Line 65. Awkward sentence regarding “the perspective is NYC”. Reword.

*We now note: The perspective is the New York City* ***Government****.*

6) Unclear how they accounted in the ROI for employee and dependent duration of enrollment. Did the costs for individuals included differ for those enrolled four months (and employee subsidy covered 4 months) versus those enrolled six months (and employee subsidy covered 6 months)? This was not clear from the methods described.

*Our approach is detailed in the main document and supporting information S1 File. In the main document we note that costs are applied monthly based on membership rates of $30 per month for Workshops and $14 per month for Digital and that NYC paid 50% of these costs for employees but did not pay for dependents. In the supplement we present the 8 step approach for quantifying savings and present two examples. In step 5 we explicitly state that we apply adjustments to savings based on participation duration (days out of 365). Operationally, we generate the savings based on a year using our assumptions and the Cawley et al estimates and then pro-rate based on enrolment duration for each participant. Note that costs accrue monthly for all participants unless they actively disenroll but savings accrue only to those with greater than 2.5% weight loss maintained for longer than 3 months. If needed we are happy to add additional details.*

7) Line 163. What did the authors do for individuals with a BMI between 29.1 and 29.9?

*Apologies but the subsequent sentence should have read ‘We linearly extrapolated savings between $0 and savings for a given BMI reduction for those with a starting BMI of 30 kg/m^2^ to impute savings for those with starting BMI values between 26 and* ***29.9*** *kg/m^2^. Thanks for catching this oversight.*

8) Line 183-185. The authors do not provide justification for why in the sensitivity analysis, they estimate savings assuming that weight loss is maintained throughout the duration of the enrollment. Why is that assumption made given the known likelihood of weight regain once the intervention ends?

*We have removed this analysis for the reasons noted.*

9) In Figure 1, the authors describe participants that were not eligible because they had an outlier for baseline height, weight, or weight change. Please add a description of what those cutoffs were, the justification for them, and how this was conducted.

*This information was added to Figure 1. We now note: 4,425 participants were ineligible due to out of range age (l.t. 18yrs or g.t. 65yrs), BMI (l.t. 26kg/m^2^) or enrolled less than two months before the end of the evaluation period.*

*We use this age range to exclude youths and because those age 65+ are Medicare eligible who is likely the primary payer of their health services. Those with a BMI below 26kg/m^2^ or enrolment duration of less than 3 months could not lose enough weigh to generate savings given our assumptions and thus were also excluded from the Savings (but not cost) analysis.*

*We further exclude 2,134 participants from the Savings analysis because they had less than two weights recorded (so could not identify a weight change),or had outlier height, weight or weight change (beyond 99^th^ percentile from NHANES 2011-2014 data):*

- *Height ≤ 58 inches or ≥ 84 inches*
- *Weight ≤ 104 lbs. or ≥ 700 lbs.*
- *Total weight loss ≥ 25%*
- *Total weight gain ≥ 25%*
- *Average weight loss per month ≥ 8.5%*

*Note that some individuals were excluded for multiple reasons.*

Results:
10) Line 247-248. This sentence is confusing.

*We now note: 84% of savings came from employees, with dependents contributing 16%.*

Discussion:
11) Additional discussion is needed on the fact that only 51% of employee and 54.1% of dependent enrollees were eligible to generate savings and related limitations.

*We now note in the Discussion section:*

*This ROI was achieved even though only 3% of employees signed up for the program and only 1/3^rd^ of those who did lost enough weight (2.5% or more) and over a long enough period of time (3 months) to generate savings. This low uptake reflects the reality that most employees are not necessarily interested in signing up for a behavioral weight management program when offered. Moreover, many of those who signed up may have found the program not to be a good fit for them and/or were not at a stage where they were ready to commit to long term weight loss. Some of these individuals are likely to reconsider this or alternative programs at a later date. Regardless, results further reveal that, due to the high costs of obesity, even moderate weight loss among a subset of participants is enough to cover the costs of the entire subsidy and generate additional savings.*

12) Additional discussion is needed on the fact that the majority of savings (73% for both employees and dependents) came from enrollees with class II or III obesity and related implications.

*We now note: It is worth noting that the majority of predicted savings (84% for employees and 80% for dependents) came from participants in the Obese III category. This result is not surprising given that this group is most expensive in terms of their annual medical expenditures as shown in Cawley et al. Although this may suggest that the city (or others) should not invest in those in lower BMI categories, this is not necessarily the case. Given that individuals tend to gain weight as they age, a dynamic analysis may show that these early interventions are effective at avoiding future costs before they occur, and thus may also show a positive return on investment. This should be an area of future research.*

13) There was a great deal of diversity related to employee types including administrative staff, healthcare providers, teachers, emergency services personnel and first responders, sanitation and janitorial services, among others. Do the authors have data related to job type, income, or any other indicator of socioeconomic status and how that may play a role in weight loss efforts of the employees and dependents?

*Unfortunately, these data are not available to us. We agree this would make for an interesting extension and should be an area of future research.* Minor comments:
There are a great deal of typos throughout the manuscript that need to be carefully checked. Some examples include:
a. Line 90. Add ‘s’ to the end of behavioral skill.
b. The word enrollment is spelled incorrectly throughout the entire manuscript (it is spelled enrolment).
c. Line 174. Comma at the end of the sentence rather than a period.
d. Line 245. Should be a space between SD and the number.
e. Line 289. Typo after analysis.

*We have revisited and updated the manuscript as appropriate, including changing spelling from British to US style.*

14) Line 211. Should be clear that it is 91% of enrolled employee and dependents that were female.

*We now note: 91% of employees and 78% dependents in the sample were female.*

**Reviewer #2**

1) Main concern [Lines 157] "We assigned zero savings to those who gained weight ..." Unlike the last two items in the next section, this definitely has a structural, biasing impact on the results and is definitely unreasonable. If someone started a program and gained 10 BMI units, that's just "no savings" to the state? Not a hefty negative savings?

*We appreciate the reviewer’s concern. We believe it is reasonable not to attribute weight gain to WW given that there is no evidence that WW promotes weight gain. However, it is potentially an overestimation to assume that all weight loss is attributable to WW given that some individuals may lose weight even in the absence of the program. To address both concerns, we now make it clear that our analysis considers weight gain and weight loss symmetrically, as suggested by the reviewer.*

2) [Data] If "Data cannot be shared publicly because of legal issues. Data are available from WW International for researchers who meet the criteria for access to confidential data.", then the data are not "available without restriction"

*Due to the contractual requirements by the City of New York we are unable to share this data without a data use agreement in place. If a requestor wishes to replicate this work, they can send a data request to Eric Finkelstein at* [*eric.finkelstein@duke-nus.edu.sg*](mailto:eric.finkelstein@duke-nus.edu.sg) *who will then initiate the process of executing the agreement. Upon approval, de-identified data will be made available to the requestor for the approved analyses.*

3) [Line 148 and beyond] The emphasis that the Cawley estimates are "causal" (note the deliberate italics in line 148), is questionable: 1) An instrumental variable approach is just a mediational analysis with some extra assumptions; without those assumptions, there's no causality. Cawley et al. do spend time addressing/justifying this point (see, for example, the passage that mentions their references 30 and 31), but (a) reference 30 is to an entire book, which is rarely a sign of argumental strength and (b) reference 31 estimates the shared environment effect at 10%, which is not nothing
   3) Lastly, to nitpick, Cawley et al. are discussing weight as an IV, not BMI. So while the argument for causality under the Cawley et al. model is stronger than the majority of other such cases where causality is invoked, this still needs some caveating by the authors.

*We agree with the reviewer’s concern. To address this we have deleted the word ‘causal’ and now simply note ‘Unlike most obesity modelling studies, their modelling strategy includes an instrumental variables approach designed to address measurement error and omitted variable bias, and thus was deemed to be the best source of data for this study.’*

4) [Lines 166-167] Item #3 in this passage seems arbitrary, unnecessary and could have a big impact on the results. Justify this choice or redo the analyses without it

*Some assumptions were necessary because Cawley does not report cost savings for weight loss greater than 20% or for those with a BMI above 45kg/m^2^. By recoding we are making conservative assumptions that would only serve to reduce the savings. Therefore, we think this is a reasonable approach. To make this clear we now note ‘Cawley et al. also does not report savings for those with greater than 20% weight loss. Therefore, those with weight loss greater than 20% were conservatively recoded to 20%.’ and ‘For those with starting BMI values above 45 kg/m^2^ we conservatively recoded the BMI to 45 kg/m^2^ and assigned savings as reported in Cawley et al. based on the amount of weight lost.’*

5) [Lines 236-238] Similarly, the fact that some dependents were deemed to not generate savings based on having "outlier data" is suspect.

*Outliers were set based on an analysis of NHANES 2011-2014 data as follows: (a) had baseline height (<58inch or >84inch), (b) baseline weight (<104lb or >700lb), (c) weight change such that BMI reduction > 25% or BMI gain > 25% or BMI reduction per month > 8.5%. These are very conservative assumptions and only serve to reduce the ROI as these individuals still generate costs, just no savings. If there is an alternative recommendation we will update accordingly.*

6) ** Lesser issues **

   [Line 65] "The perspective is NYC." This statement may make sense in the authors' discipline, but here it is a non sequitur. Please make clear what it is that you are trying to convey with this passage

*We now note the perspective as the New York City Government.*

7) [Line 82] "Dependents were free to also ..." Considering that this passage is talking about costs, and the costs for dependents is not zero (free), perhaps the authors should use a different phrasing

*We now state ‘Dependents also had the option to…’*

8) [Lines 89-90] What is meant by "discussions on behavioral skill"?

We have now expanded this section as follows: *At the start of each session participants had a private check-in with a WW coach and were given the opportunity to weigh themselves, reflect on their progress, and set actionable steps for the next week. Then the weekly group discussion began with celebrating and problem solving member’s successes and challenges over the past week. After that, a new topic and technique focused on a skill related to weight loss and behavior change was introduced.*

9) [Lines 100-102] It is unclear how (ii) and (iii) differ; the passages later on that describe these don't really clear this up either. Please clarify their distinction

*We have clarified this such that (ii) is concerned solely with baseline weight* ***values*** *that extend beyond those reported in Cawley et al and (iii) is concerned with weight* ***loss*** *beyond what Cawley et al. reported.*

10) [Lines 159-165] "for those with a BMI below 26" covers both "normal weight" and "overweight"; why is this breakpoint used?

*Because we required a minimum weight loss of 2.5% and assumed no savings for those in the healthy BMI range, the minimum BMI that could generate savings is 25.7, which we conservatively rounded to 26.*

11) ** Errata **

    [Line 36 and beyond] Make sure that "et al." is used, as this is short for et alia

    [Line 49 and beyond] Inconsistent trademarking of Weight Watchers (compare to line 7)

    [Lines 52 and beyond] Inconsistent spacing before reference numerals; see this line for a self-contained pair of examples

    [Lines 99-105 and beyond] Inconsistent capitalization of items in lists; compare (i-v) and (vi) here

    [Line 101 and beyond] Multiple instances of the typo: "enrolment"

    [Lines 108-113] This whole paragraph is a sentence fragment

    [Line 108] The parenthetical begun with "(self-reported ..." is never closed

    [Line 121] Typo: "date,(s)he" (missing a space)

    [Line 160] The period is part of the superscript in "m2."

    [Line 174] Typo: "above,."

    [Line 202 and beyond] "consort" should be "CONSORT"

*We thank the reviewer for raising these issues. They have now been addressed.*

**Reviewer #3:**

1) The manuscript “Financial Implications of New York City's Weight Management Initiative” aimed to fill an important gap about the cost effectiveness in terms of health expenditures of widespread implementation of a commercial weight loss program, WW. Unfortunately, the model includes some fatal assumptions, some highly questionable assumptions, and inadequate handling of error.

   The most substantial and fatal assumption is that individuals can only result in cost savings or no differences in costs. Individuals who have gained weight or lost less than 2.5% of weight were set to zero. However, the authors report that participants lost 6.3 to 11.6 lbs on average, but with substantial standard deviations. If weight change is assumed to be normally distributed (which the use of standard deviation would imply), then between 36% and 40% of participants would have gained weight in the sample. Claiming only success of weight losers and ignoring the costs of weight gainers necessarily biases the cost savings estimate in favor of the intervention. I note that the authors also report that 25.7% (line 232) to 27% (line 236) of participants ‘did not lose enough weight’, which indicates that the distributions are not normally distributed, and thus should not be represented by mean and SD, but still supports that a substantial proportion of the participants might have incurred cost increases rather than savings.

*As noted in Response 1 to Reviewer 2, we do not believe it is unreasonable not to attribute weight gain to WW given that there is no evidence that WW promotes weight gain. However, it is potentially an overestimation to assume that all weight loss is attributable to WW given that some individuals may lose weight even in the absence of the program. To address both concerns, we now make it clear that our analysis include a more conservative approach where we consider weight gain and weight loss symmetrically, as suggested.*

2) There is also no control group, and workplace wellness program evaluations frequently note selection effects. For instance, the Illinois Workplace Wellness Study. What would these same people have experienced without WW?

*We agree that the ideal study would include a randomized trial design and a control group and now note in the limitations section ‘Future studies should aim to look at longer term cost implications of subsidizing such programs using more rigorous randomized trial designs that are free from selection and other potential biases inherent in our study’.*

3) Some questionable assumptions are based on the use of the estimates from Cawley et al. Cawley et al. employed an instrumental variable approach to estimating the causal effect of weight loss on medical expenditure savings. The use of the Cawley estimates have limitations (Do they match the present sample? How does their approach to addressing self-report affect the measured vs self-report differences used in the present manuscript? Is the IV even appropriate?), but are not an altogether unreasonable basis to estimate changes in medical costs.

*Cawley estimates are generated from a nationally representative sample. We agree it’s not perfect but believe that the Cawley estimates provide the best source of data for conducting such an analysis. If the reviewer can suggest an alternative we will incorporate it.*

4) However, the present manuscript makes several questionable choices. First, rather than staying true to the original estimates, they interpolate values not reported by Cawley et al., including a 2.5% weight loss threshold that is lower than the 5% reported (it appears there is also a 7.5% threshold from tables that was not reported in the methods), and extrapolating below the BMI of 30 to include a greater n, despite Cawley et al. discussing the non-linearity of expenditures over BMI. Indeed, in Table 1 of Cawley, some of the changes in expenditure are not monotonic across % weight loss within some BMI categories. Second, the present authors chose to impute diabetes status because it was not included in the data available. This approach used a weak imputation, involving only estimating proportions based on probabilities of diabetes based on weight status alone (S1). This does not adjust for other key variables (e.g., the present population is predominantly women), and the performance of such an approach for expenditure modeling is not justified within the paper. More importantly, Table 1 of Cawley includes estimates that are averaged across all adults regardless of BMI status. The big concern is that Table 1 appears to be less favorable to support cost savings compared to Tables 2 and 3. Switching from Cawley’s model using all people to stratifying by diabetes status (without: Table 2; with: Table 3) means that cost INCREASES seen for individuals with a BMI of 30 who lose 20% of weight ($235 cost/yr) are instead estimated as SAVINGS for those without diabetes ($905 savings/yr) and with diabetes ($1865 savings/yr). This raises two concerns: why did the authors chose to use a weak imputation approach to estimate diabetes status when a combined model that did not need diabetes status was included in Cawley et al? And, if the models are that instable just from stratifying by diabetes status, are the estimates reliable for estimating cost savings in turn?

*The reviewer raises two concerns related to our imputation strategy. First with respect to imputing below a BMI of 30kg/m^2^, because weight loss in this range has been shown to offer some health benefits (see references in manuscript), we felt that it was appropriate to consider potential savings in this range. We agree that our imputation strategy is simplistic but given the small degree of weight loss among individuals with enrollment BMI in this category, any change to the imputation strategy would have a negligible impact on the results (these individuals generate less than $10,000 in gross savings in the revised version). Re the second point, we originally attempted to control for diabetes status due to concerns that the diabetes prevalence for NYC differs from that for the general population. However, we agree that due to the weak imputation approach, this is not justified. We have now re-estimated the results using Cawley’s table 1 which combines those with and without diabetes*

5) Some procedures were not clarified: is someone who lost 4% rounded to the 5% group, rounded down to the 2.5% group, or interpolated?

*We now note as Assumption 2: For each starting BMI, Cawley et al. presents savings for weight losses of 5%, 10%, 15% and 20%. For weight losses between these values, we estimated savings using linear interpolation.*

6) It appears that the error in Cawley estimates were not propagated forward into this model. That is, Person A in S1 is estimated to have experienced cost savings of $495.66, but what are the error bounds around that given the substantial error term in the Cawley tables? No estimate of uncertainty was provided for any of the model estimates, other than SD that appears based only on the point estimates from Cawley’s tables. The total estimated costs should also have an error term, and the SD reported for per capita values is only for the SD of mean estimates for each individual, rather than the variability compounded with the error presented in Cawley.

*We agree that the estimates we present are associated with great uncertainty. Rather than trying to statistically convey this uncertainty, which we think would be infeasible given our inability to access the raw data in Cawley et al, we note the following in the Discussion section: Results, even when limited to employees, show an ROI of 144%. However, we caution that the actual ROI could be orders of magnitude higher or lower. We could not credibly produce confidence intervals without access to the underlying MEPS data used in Cawley et al. Regardless, results provide suggestive evidence that WW, whether delivered in person or digitally across a wide variety of employees, combined with a modest employer subsidy, has the potential to generate a positive ROI. This is an important implication that should be validated in future studies.*

7) Other error terms are confusing. For instance, given the (fatal) imposition of limiting participants to cost-savings only, the estimate of $83 per capita with an SD of 385.5 implies that 41% of people saved nothing or cost-increased (again, because SD is typically used when assuming symmetry). However, the greatest loss would be $15/mo x 12 mo = $180/yr loss. Consider presenting the data in a different format (median/IQR), or clarify how losses could be greater than $180/yr.

*Because the aggregate savings and ROI are based on mean values, we believe presenting means is important. However, we agree that the presentation of SD is misleading given the skewed distribution and have now removed it in favour of presenting the median as an additional statistic. We hope the reviewer finds this change acceptable.*

8) The use of two months as a minimum length between weighings was not justified in the paper.

*Evidence suggests that health improvements from weight loss can begin within weeks; therefore we believe three months is a reasonable assumption. However, to address this concern we now note in the Limitations Section: …We further assumed that weight loss translates to savings if maintained for three months or longer, however data on this assumption are limited. Validating these assumptions and results using linked weight loss and medical claims data should be an area of future research.*

9) The inclusion of weights after the end of the evaluation was also not justified. If a weight was collected during the evaluation period, it makes no immediate sense to grab weights outside of the evaluation period.

*It is common in weight loss studies to impute for missing data. In our analysis, in some cases we do not have a weight at the end of the enrollment period but have the benefit of knowing an actual weight in the period just following the end of the enrollment period. We therefore take advantage of this information and use that weight and the last recorded weight to impute a weight at the end of the enrollment period as shown in Supplement S1. We see this as a conservative approach, given weights are likely to increase with time.*

10) Although I am highly critical of the assumptions above, I also want to highlight that the authors have included important adjustments: adjusting for inflation; including all costs for participants regardless of estimating cost savings (that is, if people were enrolled but did not contribute weights to be able to estimate costs); limiting cost savings to the months that experienced the weight loss (though this raises the question of whether two months would result in cost savings); limiting analysis (and participation) to those with overweight or greater BMI; adjusting costs by the % of expenditures covered by NYC benefits; and others. S1 was exceedingly helpful to walk through the complexities of the estimation procedures.

*We appreciate the comments and have done our best to be responsive to the concerns raised. We are happy to make additional changes as requested such that the results fairly represent the findings of this research*

11) Other concerns:

    The data availability statement does not match PLOS guidance. The answer should be ‘no’ followed by an explanation in the ‘describe where the data may be found…’ field. There, the authors should clarify exactly how data can be acquired, what is meant by ‘meet criteria for access to confidential data’, and who to contact.

Due to the contractual requirements by the City of New York we are unable to share this data without a data use agreement in place. If a requestor wishes to replicate this work, they can send a data request to Eric Finkelstein at [eric.finkelstein@duke-nus.edu.sg](mailto:eric.finkelstein@duke-nus.edu.sg) who will then initiate the process of executing the agreement. Upon approval, de-identified data will be made available to the requestor for the approved analyses.

12) The abstract is not immediately clear that per capita savings for employees actually means savings for the employer (NYC).

*This change has been made.*

13) Several places slip from indicating PREDICTED savings to implying actual savings.

    Enrollment vs enrolment spelling consistency.

    The diagram is a STROBE, not CONSORT, diagram because this is an observational study.

*These changes have been addressed.*

**Reviewer #4:**

1) This paper describes simulated medical cost savings due to weight loss of participants in a Weight Watchers program. The paper is motivated by trying to establish cost savings to employers by subsidizing membership fees to the Weight Watchers program. The paper is in general well written and clear to follow. That being said the analysis is based on some broad assumptions and there are some key missing features which it would be helpful to include.

*We thank the reviewer for the positive comments and do our best to address the concerns as noted below.*

2) Introduction –
   The authors use the values of $260 on the low end and $12,548 on the high end of medical expenditure savings for weight loss taken from Crowley et al. The authors do not mention the high standard errors with these measurements or the overall analysis of the MEPS data that is conducted by Crowley et al showing significantly lower savings. Both should be addressed.

   *In response to Review 3, we now use Cawley’s Table 1 to generate the savings without effort to impute for diabetes status. As this Table uses the full MEPS data, results are generally more precise than those reported in his Tables 2 and 3. However, results are still measured with error. Rather than trying to statistically convey this uncertainty, which we think would be infeasible given our inability to access the raw data in Cawley et al, we note the following in the Discussion section: “…It is worth noting that these results represent the best estimates of the costs, savings, and ROI to the city of New York for subsidizing the program. However, they are estimated with great uncertainty that we were unable to quantify due to the limitations above. Whether these results would generalize to other worksites should be an area of future research.”*
3) If the state is not subsidizing dependent enrollment in WW what is the states roll in providing this service to dependents?

   *Aside from allowing dependents to enrol through the NYC portal, the city provides no role in the provision of WW but reaps the benefits of any weight losses and medical savings achieved. As we present results separately by employees and dependents, readers are free to consider only those savings they deem relevant.*
4) The authors claim on line 50-52 that WW produces weight loss of approximately 5% and is cost effective. Is this an average or estimated weight loss percentage? Additionally, the reference used for the claim of cost effectiveness (29) is based on a study of prediabetic subjects not the general population. This should be addressed by the authors.

*We simplified this sentence as follows: WW provides a weight management program available in person and /or digitally. The WW program has been shown both to produce clinically meaningful weight loss [9-15] and be cost effective [16].*

*We updated the cost-effectiveness reference to include the following studies from the general population of obese adults:*

[*Incremental cost-effectiveness of evidence-based non-surgical weight loss strategies.*](https://www.ncbi.nlm.nih.gov/pubmed/30677252)

*Finkelstein EA, Verghese NR. Clin Obes. 2019 Apr;9(2):e12294. doi: 10.1111/cob.12294.*

5) Line 54 The authors state that the goal of the study is to quantify the total and per capita net annual medical expenditure savings. This appears inconsistent with the Authors’ Assumption #9.

*If this comment is in regard to our sensitivity analysis, where we estimate savings assuming that weight loss is maintained throughout the duration of enrolment, that assumption has been removed from the manuscript. If the author was raising another concern, we would appreciate clarification.*

6) Sample
   Is the 430,000 the number of employees that meet the study’s criteria or all NYC employees?

   *This is all NYC employees eligible to enrol based on NYC eligibility (not WW) criteria (i.e., full time staff eligible for benefits)*
7) The authors do not state why the cutoff BMI for eligibility is 25 kg/m2 or if participants with a starting BMI between 25-26 are included in cost savings or city cost calculations.

*As noted in response 15 to Reviewer 3, because we required a minimum weight loss of 2.5% and assumed no savings for those in the healthy BMI range, the minimum BMI that could generate savings is 25.7 kg/m^2^, which we rounded to 26. Therefore, those below this value were not included in the cost or savings analysis.*

8) Do other dependents include children? Are medical expenditure savings the same for all age ranges?

*As noted in supporting information S1 File, analyses are limited to eligible adults between the ages of 18 and 65. The medical expenditure savings are based on the Cawley et al. results, which are not age dependent.*

9) Are there age cutoffs for the study? In Crowley et all, the estimates are for a population of adults between the ages of 24 and 64 years with biological children between the ages of 11 years (132 months) and 20 years (240 months). How does this compare to the sample in this study?

*As noted above, analyses are limited to eligible adults between the ages of 18 and 65, with a mean age of 45. Although Cawley does not report a mean age, we suspect it is very close to this level given that is the midpoint of their age range.*

Intervention.

10) What is behavioral skill?

*This term has been replaced with ‘behavior change strategies’.*

Measures

11) Are the authors extrapolating partial year savings to full annual savings? This is not clear.

*Partial year savings are not extrapolated. If participation duration of a participant was less than a year, the predicted savings for the participant is adjusted for this duration. Therefore, a participant whose participation duration was 6 months will be allocated 50% of the annual savings estimated by Cawley et al.*

Data

12) The authors do not explain how they impute diabetes prevalence.

*This imputation is no longer part of the analysis as we now use Cawley’s Table 1 to estimate savings.*

Analysis

13) Line 121 - The authors state that if a participant did not have a disenrollment date they are assumed to be enrolled till the end of the evaluation period. Are there weight measurements for all these subjects at the end of the evaluation period to demonstrate that the weight loss was maintained during this time? Otherwise, what indications do the authors have that indicate weight loss besides the participants continuing to pay for their portion of program cost.

*We make this assumption to ensure we appropriate apply costs during active periods of enrollment. However, as stated in the Methods section under Assumption 6: All predicted savings are assumed to accrue immediately* ***and terminate after the last weigh in****. In other words, we do not allow time for savings to accrue nor do we allow for residual benefits.*

14) The paragraph starting on line 125 is confusing. If the evaluation period is between June 2016 and May 31, 2017 why are there weight measurements from July and August 2017? How many subjects did this occur with and were they still enrolled in the WW program at the subsidized rate from the end of May until their final weight measurements? If not, how do the authors attribute any weight loss during this time to the WW program?

*Although the analysis period ends May 31, 15% of participants remain enrolled after this date. We use the weights in the subsequent two months simply to help us impute a final weight at the end of the enrolment period for those who remain enrolled as detailed in the supporting information S1 File. Note that taking advantage of these weight is conservative given that people are more likely to gain than lose weight over time.*

15) Line 131 – For participants with a duration of zero, are the costs incurred by the city for these participants included in the cost savings calculation?

*Yes, these participants contribute costs but do not contribute to savings.*

16) Line 135 – The authors state that if participants have an additional weight recorded in the three months after the evaluation period their final weight is a weighted average of the final two weights. How is this weighted average calculated and how many participants did this include?

*This is shown in the example for Person A in supporting information S1 File. This included 15% (2,300 participants out of 14,946) of participants included in the analysis sample.*

17) Were these participants still enrolled in the program at the subsidized rate from the end of May until their final weight measurements, if not how is weight loss after the end of the evaluation period attributed to the Weight Watchers program.

*Yes. These participants are still enrolled at the subsidized rate.*

18) Assumptions - While briefly discussed, the assumptions the authors make to extrapolate outside published literature on cost saving due to weight loss are very broad. These assumptions should be addressed up front in the paper as they cast doubt on the validity of the cost savings analysis.

*Rather than address this up front, we now provide a greater discussion of the assumptions in the limitations section, which we believe is the appropriate place for such a discussion but will move this up if the Editor requests. Note that we have also made several revisions to our assumptions in efforts to address reviewer concerns.*

19) Assumption 1 - Extrapolating for cost saving for under 5% weight loss especially at lower starting BMI does not appear to be supported by reference 9 and 10.

*We have removed these references for the avoidance of doubt.*

20) Assumption 2 – Extrapolating cost savings for subjects under a starting BMI of 30, especially for weight loss of under 5 percent is a large assumption that should be addressed up front. A 5’7” male with a weight of 170lbs has a BMI of 26.62. A 2.5% reduction in weight is only 4.25lbs which is within established values for normal daily weight fluctuation.

*It turns out that this extrapolation had a negligible impact on the results. Individuals with a starting BMI below 30 had only modest weight loss and generated less than $10,000 in gross savings out of a total of over $3m (based on the revised estimates). We believe it is worth keeping this information in the paper for readers to see the small effect that this BMI range has on the gross and net savings.*

21) Cost savings calculations should be reported without extrapolating below a starting BMI of 30 and without extrapolating below 5% weight loss for comparison.

*We present the information in the tables separately by starting BMI category and by degree of weight loss so this information is included in the tables.*

22) Assumption 3 – The author gives no explanation for this recoding of the data.

*We now note ‘Cawley et al. does not report savings for those with greater than 20% weight loss or BMI values above 45 kg/m^2^. Therefore…’*

23) Assumption 4 – The authors state that only participants with more than one weight recorded were eligible to contribute to savings. Did these individuals contribute to the city’s cost for subsidizing the program?

*Yes. All those who were enrolled contributed costs during the period of enrollment regardless of whether they provided weight data.*

24) Assumption 8 – The authors appear to treat all medical expenditures as equal regarding cost. Do the authors take into account different costs for covered vs not covered procedures?

*We do not have access to that level of detail. As noted in Assumption 8, the City of New York’s base health plans covers 74.7% of medical expenditures referenced in the Cawley model. Therefore, for lack of a better alternative, we assumed this percentage of savings accrues to the city.*

25) Assumption 9 – The authors assume that weight loss is immediate for all participants which is an unreasonable assumption. Additionally, the authors assume that weight loss is maintained after an individual’s final weight in. Is there any evidence that this is actually the case?

*As noted in Assumption 6, all predicted savings are assumed to accrue immediately* ***and terminate after the last weigh in****. We do not assume that savings are maintained beyond the final weigh in. We agree that the assumption that savings accrue immediately is overly optimistic, but we also believe that assuming residual benefits cease immediately after the last weigh in is overly pessimistic. We now state these assumptions explicitly in the limitations section: We further assumed that weight loss translates immediately to savings if maintained for three months or longer, but ceases immediately after the last weigh in. However, data on this assumption is limited. Validating these assumptions and results using linked weight loss and medical claims data should be an area of future research.*

26) Sample Characteristics
    The authors state that 91% of employees and 78% of dependents were female. Are there any differences between the cost savings due to weight loss for males vs females?

*This is possible but the Cawley estimates are not gender specific therefore we did not attempt to present estimates separately by gender.*

Duration

27) The authors state that 47.8% of employees and 45.1% of dependents did not generate cost savings. Did these participants contribute to the cost NYC incurred in the cost savings calculations?

*Yes. Costs were generated for all participants for all periods of enrollment.*

28) Limitations
    The authors state that self-reported weights for digital participants (42% of overall sample) were similar to other online programs that relied on measured weight. What programs are the authors using for comparison and what criteria is used to determine similarity of measurements?

*We have removed this reference because we no longer are convinced that argument is appropriate. Self-report bias could occur regardless of whether or not results from our program are similar or different to measured results from other programs. We now simply state: Other limitations are that only self-reported weight was available for Digital participants.*

29) The authors do not acknowledge or discuss possible increase in medical expenditures due to weight loss. i.e. An individual that needs a hip replacement but can not get the surgery until losing weight. An individual in this situation would incur increased medical expenditures due to weight loss rather than cost savings. This should be discussed as a limitation of this analysis.

*This may occur but presumably this is built into the Cawley estimates thus we do not believe it is a limitation of our analysis. Moreover, whereas that is likely to occur after large weight losses among very high BMI individuals, such as after bariatric surgery, it is less likely to result from behavioural weight loss programs such as WW that tend to target individuals in the BMI 25 to 40 kg/m^2^ range.*
