# Supplementary material for: Financial implications of New York City’s weight management initiative
Source: PLoS One. 2021 Feb 11;16(2):e0246621. doi: 10.1371/journal.pone.0246621 (PMC7877753; doi:10.1371/journal.pone.0246621)
Supplement: Attachment — Submitted filename: Response to Reviewers.docx [file pone.0246621.s006.docx]

**Reviewer Comments**

A) **Reviewer # 1** – No additional comments
B) **Reviewer #2** – No additional comments
C) **Reviewer #3:**
1) I appreciate the work the authors put into addressing the many reviewer comments. Despite my critiques, I am truly interested in seeing this analysis come to fruition, but the description of the approach is not clear enough that I could replicate it (sharing code may help), some assumptions still seem inappropriate, statistical summaries are still missing, and some artifacts/controls are not addressed.

*We are grateful that the reviewer is keen to help us further improve the manuscript to the point of publication. We recognize that our approach is not perfect, and admittedly would have done things differently if starting from scratch, but we believe the current approach, with the improvements noted below, is highly defensible and worthy of publication.*

*We had thought our approach was transparent from point of initial submission both in the methods section and via our supplemental appendix. We also made efforts to further clarify our approach in the first revision. In this iteration we have made additional changes to address specific reviewer concerns, as discussed below. We also now include the stata code as an additional supporting information S2 File. STATA Code for the reviewer. We will gladly make this code available upon request or as part of the publication should the editor request. If the reviewer has additional specific recommendations for how we can further clarify our approach we are happy to incorporate those as well.*

2) My understanding of what the authors would like to accomplish is essentially to take an individual at baseline, map them to the estimates in Figure 1 of Cawley, then at the end of the period map them to Figure 1 again. Then, for each individual, subtract the estimated medical costs at time 1 from time 2 and get a change (which the authors refer to as ‘Savings’, even when negative).

*In hindsight and assuming we could get access to the underlying regression equation that generated Figure 1, this might have been our preferred approach. However, as our initial submission focused on weight loss only, and we estimated separate estimates for those with and without diabetes, our starting point was Cawley et al. Table 2 and 3 that showed predicted change in total annual medical expenditures ($US) from the instrumental variables model for those without (Table 2) and with (Table 3) type 2 diabetes.*

*Based on feedback from the prior round of review we abandoned efforts to incorporate diabetes status and instead focused on Cawley et al. Table 1 which presents estimates for the full sample. What we did was put each WW participant in the correct row (row x) of Table 1 based on their starting BMI, then the correct column (column y) based on how much weight (BMI %) they lost and then pulled the savings from that cell (x,y) as the annual savings. We further adjust the savings based on the duration of the weight loss (i.e., if the duration is six months we give them half of Cawley et al. annual savings).*

3) However, because Cawley et al. only report numerical values for the starting BMI in Table 1 with % changes in weight, the authors are imputing across these % change categories (5%, 10%, 15%, and 20% weight change). The authors use linear imputation to estimate between categories, and extrapolation to go between 5% and 0%, despite substantial non-linearity demonstrated in Figure 1 of Cawley, in addition to extrapolating below BMI of 30. The simplest approach would just be to contact Cawley et al. and ask for their parameter estimates, rather than complicating matters with imputation strategies.

*We agree that in hindsight contacting Cawley and asking for the parameter estimates might have been preferred, but we believe our imputation/extrapolation approach is completely defensible. Below we reproduce Cawley et al. Figure 1, but add the dark blue line segments between each 5 unit BMI value. What can be seen from the Figure is that despite substantial non-linearities across the entire BMI spectrum as the reviewer notes, BMI is largely linear within each 5 BMI unit line segment between 25 and 40, thus justifying our imputation approach. In fact, only between BMI 35 and 40 do we clearly see the non-linearity coming through and given that the curve falls below our line segment, it means that our assumption of linearity within this segment is conservative (we assign slightly lower savings for a reduction from, for example, BMI 40 to 37 then Cawley et al. curve would predict). For other ranges his curve and our line overlap. For these reasons, we believe our approach for estimating savings is reasonable.*

4) Regardless, it is unclear how the authors are implementing this approach, particularly for weight gain situations. The authors now say they use weight gain and weight loss estimates ‘symmetrically’ (in the response to reviewers) for costs and savings. ‘Symmetric’ is not necessarily correct because someone gaining 5% starting at BMI 30 would not be the same cost/savings as someone losing 5%.

*We appreciate the reviewers comment and have clarified our approach. We now under Assumption 1:*

*Cawley et al. only reported savings for those with weight loss of 5% or greater. We linearly extrapolated savings for all those with BMI reduction (i.e., weight loss) between 2.5% and 5%. For example, if an individual had a 2.5% reduction in BMI then she would receive medical cost savings of half of the corresponding value in the 5% BMI reduction column in Cawley et al. Table 1. Clinical evidence suggests that weight loss as little as 2.5% generates clinical health improvements [4-6] thus, we deemed it reasonable to apply cost savings for weight losses of this magnitude or greater, although we explore the implications of this assumption in the sensitivity analysis. For weight losses smaller than 2.5% no savings were assumed.*

*And under Assumption 6:*

*To minimize risks of bias, we apply the estimates in Cawley et al. Table 1 in reverse for those who gained weight. For example, someone with a starting BMI of 33kg/m^2^ who lost 5% of their baseline weight would have a predicted savings in annual medical expenditures of $288 (in $2010). If a different individual with the same starting BMI gained 5% of their baseline weight we apply the $288 as a cost, not as a savings. This conservative approach addresses concerns due to omitted variables, mean reversion [20], and other potential sources of bias.*

*We further note in the limitations section:*

*Although we conservatively applied costs for those who gained weight, we assumed that BMI increases of a given percentage have the same effect on annual medical expenditures, in absolute terms, as BMI reductions. In reality, risks of chronic disease and medical expenditures increase non-linearly with increasing BMI. Therefore, our cost increases for those who gained weight may be underestimated. This is further exacerbated by applying zero costs for overweight individuals who gained weight. However, given only 15% of our sample, including overweight participants at baseline, gained weight and average weight gain was only 5.7lbs, varying these assumptions is likely to have little impact on the resulting ROI estimate.*

5) The extrapolation below 5% is still potentially inappropriate because Figure 1 shows that the nadir of the costs appears to be around 27-28 (difficult to estimate by eye). The assumption that savings starts at 0 therefore is not necessarily appropriate, as reinforced by the negative values in Cawley table 1 that show increased costs when participants of BMI 30 lose 20%. This negative value is ignored by the authors (e.g., Line 58 says a low of savings of $69, rather than a COST of $235). Similarly, the curvilinear nature of Figure 1 indicates that estimates of change will be over or underestimate depending on weight gain or loss for every individual, and this becomes more substantial as BMI increases. Although the authors justify using a 2.5% weight change cutoff, that is not the model they are proposing. If they assert that Cawley et al. have demonstrated continuous, causal estimates of changes in expenditure, the models are continuous over the full range, and thus should include even modest changes, rather than creating discontinuities. These discontinuities may be substantial at higher BMIs (e.g., $5k for a BMI of 45 losing 2.5%).

   *Some of the issues the reviewer raises here were addressed in the prior responses. However, two issues remain, which we address in turn.*

***Extrapolating weight losses between 0 and 2.5%:***

*Even though Cawley’s continuous model would allow for estimating savings for weight change below 2.5%, we do not believe the clinical evidence provides justification for such an assumption. Therefore, we believe it is appropriate to assume zero savings (or costs) for weight change below this percentage. We note in the manuscript: Clinical evidence suggests that weight loss as little as 2.5% generates clinical health improvements [4-6] thus, we deemed it reasonable to apply cost savings for weight losses of this magnitude or greater.*

***Extrapolating weight losses below 30 BMI.***

*This is another excellent point raised by the reviewer and one we did not adequately consider in prior versions. Cawley et al. state in the manuscript that the nadir on costs occurs at a BMI of 25 kg/m^2^, but the reviewer rightly points out that expenditures appear unaffected by changes in BMIs within the overweight range and that weight losses that generate BMI values below 25kg/m^2^ may lead to weight gain.*

*Given our current methods, we believe the most straightforward and fully defensible approach is to apply zero savings from weight loss to anyone whose starting BMI is below 30 kg/m^2^(i.e., not extrapolating to BMI values below those listed in Cawley et al. Table 1). We now note under Assumption 3: We assumed $0 savings for those with a starting BMI in the overweight range due to lack of evidence that weight loss translates into reductions in medical expenditures for this BMI category.*

*We also note in the limitations section, as stated above ‘... Therefore, our cost increases for those who gained weight may be underestimated. This is further exacerbated by applying zero costs for overweight individuals who gained weight. However, given only 15% of our sample, including overweight participants at baseline, gained weight and average weight gain was only 5.7lbs, varying these assumptions is likely to have little impact on the resulting ROI estimate.*

6) The authors state "We believe it is reasonable not to attribute weight gain to WW given that there is no evidence that WW promotes weight gain." This is an unreasonable assumption here. Ignoring that "absence of evidence is not evidence of absence", and that every weight loss program has some concern of rebound weight gain, my concern is not actually of attributing weight gain to any particular cause. It is a mathematical/statistical concern. If the authors were to synthesize a random sample with a mean actual weight change of zero, and select only those with randomly lower weights, and then attributed those apparent ‘weight losses’ to their program, they would get an apparent benefit of the program, even if they set all of those who had an increase in weight to zero. Thus, the weight gain must be included, and I am glad to see that it appears now to be, though again exactly how is unclear.

*We appreciate the reviewer’s concern and have now included medical costs for weight gain and provided additional clarity for our approach as described in response 4 above.*

7) A bigger concern is that there is no control group. Saying that the program is cost effective indicates the program is having an effect.

*We appreciate the authors concern but note that the evidence for the effectiveness of WW is already well established via high quality randomized trials, as we cite in the introduction. That is not the focus of this analysis. Rather our goal is to use real world evidence to simulate the potential savings from this evidence based program. We agree that the ideal study would have a control group and make this point clear in the manuscript, however, we still maintain that this manuscript provides important information, despite the limitations.*

8) What proportion of individuals who selected the program would have spontaneously lost that degree of weight anyway? The authors’ model presumes all success is attributed to WW, which may be true, but remains unproven.

*We appreciate the reviewer’s concern but maintain that results of real world evidence studies are important to publish despite their limitations. However, we agree that these studies need to be transparent and limitations noted. We now state in the limitations section: Importantly, although these results represent the best estimates of the costs, savings, and ROI associated with the subsidized program, we cannot say the program caused the savings. This results, because, in the absence of the subsidy, some employees would have made efforts to lose weight either by joining WW and paying on their own, or in myriad other ways, and some would have been successful. Therefore, these results may represent an upper bound of the potential savings resulting from the subsidized weight management program.	…. Future studies could improve on these estimates by using linked data on weight change and changes in medical expenditures using more rigorous randomized trial designs that are free from selection and the other potential biases and that can incorporate both parametric and non-parametric sensitivity analyses….*

9) The authors mention that 80% of ‘savings’ came from the obesity III+ category, which is the most highly variable portion of Cawley. Worse still, this is the portion of the distribution who are more likely to experience improvements by regression to the mean. Thus, any spontaneous contraction of this end of the distribution downward would give the appearance of substantial effectiveness of WW, and thereafter multiplied by medical expenditure estimates (c.f., <https://pubmed.ncbi.nlm.nih.gov/31552422/>). Perhaps published approaches to try to account for regression to the mean would address the issue for this manuscript at least in part?

   *We address variability in our response to Comment 11 below but remain unconvinced that spontaneous contraction or regression to the mean is a significant concern here. The referenced manuscript suggests that misattribution of RTM typically occurs when investigators restrict analyses in a segment of the sample above or below the population mean to determine pretest to posttest intervention changes and do not utilize a control group. In our case, we include all those with BMI values above 30 kg/m^2^ at baseline so have not restricted the sample to the extreme ends of the distribution (at least not for NYC). It is true that most of our savings comes from the Obese III group, but this is to be expected as this is the group that has the highest health risks at baseline and therefore the most potential for savings due to weight loss. Using the authors example of the PEACH child overweight study, one way to test for RTM is via longitudinal data of ‘controls’. With respect to Obese III adults, longitudinal data reveals that, in the absence of an intervention, they do not tend to lose weight or ‘regress to the mean’. In fact, as shown in the Figure below, reproduced from Young Adult Weight Trajectories Through Midlife by Body Mass Category* [*Rahul Malhotra*](https://pubmed.ncbi.nlm.nih.gov/?term=Malhotra+R&cauthor_id=23408493) [*^1^*](https://pubmed.ncbi.nlm.nih.gov/23408493/#affiliation-1)*,* [*Truls Ostbye*](https://pubmed.ncbi.nlm.nih.gov/?term=Ostbye+T&cauthor_id=23408493)*,* [*Crystal M Riley*](https://pubmed.ncbi.nlm.nih.gov/?term=Riley+CM&cauthor_id=23408493)*,* [*Eric A Finkelstein*](https://pubmed.ncbi.nlm.nih.gov/?term=Finkelstein+EA&cauthor_id=23408493)*, Obesity (Silver Spring). 2013 Sep;21(9):1923-34.  doi: 10.1002/oby.20318., 68% of those who enter adulthood with a BMI in the Obese III range will continue to gain weight as they age at least through midlife. Moreover, even for the 32% who lose weight, average annual weight loss is small, less than one pound per year. As such, whereas we agree that our analysis is subject to several potential biases due to lack of a control group, something we readily acknowledge and address in the manuscript, we do not believe additional efforts are needed to specifically address RTM concerns.*

10) The authors still present no estimate of variability in the estimates. The authors replace SD estimates (with their distributional problems) with medians, and the reader is still left wondering what the distribution of savings/costs are by starting/ending BMIs or changes in BMIs.

*We address the variability issue in response to Comment 11. In the prior submission we presented Gross savings by Enrolment BMI (Table 3) and by BMI reduction (Table 4). We now present, in the supporting information S1 Table, net savings for these same categories. As discussed in Comment 11, we also present Gross and Net Savings and ROI for a series of sensitivity analyses in Table 6. We hope this addresses the reviewer concerns but will include additional analyses should the reviewer or editor request.*

11) The assertion that to “statistically convey this uncertainty [is] infeasible” is not necessarily true. Cawley et al. present variability estimates for each of the percent categories of weight change, which could be used for a resampling based approach for error estimates, for instance.

*The reviewer is correct that a statistical approach to uncertainty may be feasible. However, we propose a non-parametric approach that is common in economic evaluations. We present several one-way sensitivity analyses that vary key parameters of interest. For this analysis, these parameters include 1) minimum weight loss to generate cost savings, 2) minimum duration that weight loss must be maintained, and 3) costs savings for any given starting BMI/weight loss. Therefore, in addition to our primary estimates, we now include gross and net savings and ROI under the following scenarios:*

1) *Minimum weight loss to generate expenditure savings is 5% (as opposed to 2.5%)*
2) *Minimum duration to generate expenditure savings is 6 months (as opposed to 3 months, and*
3) *Costs are 50% of what is reported in Cawley et al. Table 1.*

*We believe this approach will give the reader a sense of the variability in the estimates and addresses the reviewers concerns.*

*In the limitations section, we also note:…These estimates are also associated with great uncertainty. In efforts to gauge the influence of key parameters on the estimates, we present savings and ROI estimates both by applying stratifications that allow for determining which population subsets are most influential in the ROI calculation and by including one-way sensitivity analyses of key input parameters. Even in our most conservative scenario of savings reduced by 50%, the forecast ROI remained positive. Future studies could improve on these estimates by using linked data on weight change and changes in medical expenditures using more rigorous randomized trial designs that are free from selection and the other potential biases and that can incorporate both parametric and non-parametric sensitivity analyses…*

12) The instability of the authors’ approach is highlighted by the fact that the ROI from the first submission of this manuscript to this version almost doubled the estimated ROI, despite indicating several changes that were supposed to be ‘conservative’ (including saying they included costs from weight gain). How did this happen? How did employee mean savings increase by 50%, and net savings with dependents more than double?

*The reviewer may recall that the first submission relied on a weighted average of savings presented in Cawley et al. Table 2 (non-diabetics) and Table 3 (diabetics), with the weights representing the probability of the participant having diabetes. Based on the reviewer feedback, the analysis in all subsequent versions is based on estimates from Cawley et al. Table 1, which relies on the full underlying MEPS data. Because Cawley’s modelling approach is non-linear, this had a large impact on the estimates. Let me give an example to demonstrate how this change is driving the change in the savings estimates.*

*Example: Participant enrollment BMI = 40 kg/m^2^, assumed probability of diabetes from NYC HANES 2013-14 data set for participant with enrollment BMI = 40 kg/m^2^ is 0.296. BMI reduction= 5%.*

*From Cawley et al. Table 1, the estimated medical saving for participant with enrollment BMI 40kg/m^2^ and a 5% BMI reduction is $2137.15 ($2010). From Cawley et al. Table 2, the estimated medical saving for participant with enrollment BMI 40kg/m^2^ and a 5% BMI reduction for participant without diabetes is $643.39 ($2010). From Cawley et al. Table 3, the estimated medical saving for participant with enrollment BMI 40kg/m^2^ and a 5% BMI reduction for participant with diabetes is $2122.72 ($2010). Therefore, the weighted average medical saving from Table 2 and Table 3 estimates = 0.296*2122.73 + (1-0.296)*643.39 = $1081.27. This is almost half the savings estimated from Table 1 for the same participant.*

*Therefore, Cawley et al. Table 1 estimates of savings are driving the higher savings estimates than the weighted average of savings from Cawley et al. Table 2 and Table 3.*

*We acknowledge that our estimates are associated with great uncertainty but believe this is adequately address through our transparent approach, inclusion of sensitivity analyses with costs at 50% of Cawley’s base case values, and via raising the concern in the limitations section as noted above. We hope you agree this is sufficient to let readers decide on the veracity of our approach and resulting estimates.*

D) **Reviewer #4: Comments to the Author**

   1) The authors have made quite a substantial effort to address my previous comments and those of the other reviewers. Their responses are well balanced and have contributed to my understanding of the manuscript and I think that it is much improved. The authors have also made efforts to tone down their clams. There are still a few points that I feel need to be addressed.

*We appreciate the positive comments and address the remaining points as noted below****.***

2) In the introduction the authors start discussing a minimal weight loss of 2.5% and use a BMI of 26 as their lower cutoff. When discussing the range of savings, the authors start the low end of the range at a BMI of 30 and weight loss of 5%. If the authors want to use weight loss of 2.5% and use a BMI of 26 as their lower cutoff they should start the savings range there.

*Thanks for the comment. Cawley et al. paper did not consider those with less than 5% weight loss or those with BMI’s below 30 kg/m^2^. That is why we had to stick with these values. To clarify, we now note: Cawley et al [8] used nationally representative data and econometric analyses to estimate medical costs savings resulting from weight loss of 5% or greater for those with BMI values ranging between 30 and 45 kg/m^2^. He reports that, even with just 5% weight loss, the estimated annual savings in medical costs are $US69 (in $2010) for those with a starting BMI of 30 kg/m^2^, $US528 for those with a starting BMI of 35 kg/m^2^, and $US2,137 for those with a starting BMI of 40 kg/m^2^. In most cases savings are predicted to increase with greater weight loss.*

3) The authors slip from indicating predicted savings to imply actual cost savings in lines 358 to 359 stating “As a result the program generated a positive return on investment for NYC.”

*We agree. This sentence was not needed and has been removed.*

4) In assumption 3 the authors state that individuals with BMI over 45 were recoded to a BMI of 45. In assumption 4 the authors state that those with greater than 20% weight loss were recoded to 20%. The authors should report how many participants were recoded in each scenario.
1. *We now note: For the less than 1% of participants with starting BMI values above 45 kg/m^2^ (n=858) we conservatively recoded the BMI to 45 kg/m^2^ and assigned savings as reported in Cawley et al. based on the amount of weight lost.*
2. *….* *Cawley et al. also do not report savings for those with greater than 20% weight loss. Therefore, for those few cases with weight change greater than 20% (n=58 for weight loss, n=7 for weight gain), we recoded the change to 20%.*
5) On line 397 the statement “This study provides suggest evidence that the program…” should be reworded.

*We have reworded this sentence to state: This study shows the potential for modest subsidies of evidence based, commercially available weight management programs, whether delivered in person or digitally, to generate a positive return on investment.*

6) The quote from the paper used to respond to Reviewer 3, item 2 does not match what is in the paper.

*We apologize for this oversight. We have ensured that the reviewer responses and manuscript are now in concert.*

7) When responding to Reviewer 3 item 6 the authors state that they caution that the actual ROI could be orders of magnitude higher or lower. This caution does not appear in the paper.

*Thanks for raising this point. We have addressed Reviewer 3’s concerns as noted Response 8 to Reviewer 3.*
