# Supplementary material for: Financial implications of New York City’s weight management initiative
Source: PLoS One. 2021 Feb 11;16(2):e0246621. doi: 10.1371/journal.pone.0246621 (PMC7877753; doi:10.1371/journal.pone.0246621)
Supplement: Attachment — Submitted filename: Response to Reviewers.docx [file pone.0246621.s007.docx]

Reviewers' comments:

Reviewer's Responses to Questions

Comments to the Author

1. If the authors have adequately addressed your comments raised in a previous round of review and you feel that this manuscript is now acceptable for publication, you may indicate that here to bypass the “Comments to the Author” section, enter your conflict of interest statement in the “Confidential to Editor” section, and submit your "Accept" recommendation.

Reviewer #3: All comments have been addressed

Reviewer #4: (No Response)

2. Is the manuscript technically sound, and do the data support the conclusions?

The manuscript must describe a technically sound piece of scientific research with data that supports the conclusions. Experiments must have been conducted rigorously, with appropriate controls, replication, and sample sizes. The conclusions must be drawn appropriately based on the data presented.

Reviewer #3: Yes

Reviewer #4: Yes

3. Has the statistical analysis been performed appropriately and rigorously?

Reviewer #3: Yes

Reviewer #4: Yes

4. Have the authors made all data underlying the findings in their manuscript fully available?

The [PLOS Data policy](http://www.plosone.org/static/policies.action#sharing) requires authors to make all data underlying the findings described in their manuscript fully available without restriction, with rare exception (please refer to the Data Availability Statement in the manuscript PDF file). The data should be provided as part of the manuscript or its supporting information, or deposited to a public repository. For example, in addition to summary statistics, the data points behind means, medians and variance measures should be available. If there are restrictions on publicly sharing data—e.g. participant privacy or use of data from a third party—those must be specified.

Reviewer #3: No

Reviewer #4: No

5. Is the manuscript presented in an intelligible fashion and written in standard English?

PLOS ONE does not copyedit accepted manuscripts, so the language in submitted articles must be clear, correct, and unambiguous. Any typographical or grammatical errors should be corrected at revision, so please note any specific errors here.

Reviewer #3: Yes

Reviewer #4: Yes

6. Review Comments to the Author

Please use the space provided to explain your answers to the questions above. You may also include additional comments for the author, including concerns about dual publication, research ethics, or publication ethics. (Please upload your review as an attachment if it exceeds 20,000 characters)

Reviewer #3:

I thank the authors for addressing my many comments. This revision clarified multiple aspects of the study and while we could argue about assumptions ad nauseum, the authors have made their assumptions and processes clear to the reader. The clarity of assumptions, including the sensitivity analyses, give the reader an idea of the ramifications of various assumptions, which is critical for a paper of this sort. Thank you.

*We thank the reviewer for the positive comment.*

I few minor points the authors should consider, or would hopefully be caught in proofing:

-The Roman numerals in the Measures section repeats iii; all subsequent ones should be renumbered.

*Thank you for pointing this out. We have now renumbered the Roman numerals in the ‘Measures’ section to rectify this mistake.*

-Line 299: a distracted (or nefarious) reader may misinterpret the phrase 'based on the mean values' to be selecting means over medians to inflate the total estimates, whereas the summaries are just sums of savings (equivalent to multiplying means by n). I recommend removing "Based on the mean values".

*We agree that the wording can be misinterpreted. We have removed the words “Based on the mean values” and the sentence now reads “Total predicted savings for employees was $1,940,050 and $582,479 for ‘Workshops’ and ‘Digital’ respectively.”*

-Tables may benefit from legends/footnotes so they can better stand on their own for interpretability.

*We believe the tables are clear as presented but will add additional footnotes if the editor requests.*

-Line 434: perhaps rephrase "we do not see this as a concern" as it might read as though the authors are not concerned about the people mentioned.

*Thank you for pointing this out. We have removed this part of the sentence and simply note that:* “*However, for those with starting BMI values above 30 kg/m^2^ in our data, only 0.05% lost enough weight such that their final BMI was below 25 kg/m^2^ at follow up.”*

-Line 452+ regarding addressing 'great uncertainty': I appreciate the different models. Perhaps in the methods and here the authors could briefly but explicitly make it clear that rather than focusing on estimating point-estimate error bounds based on error assumptions (e.g., using the variability in Cawley) the authors chose to provide sensitivity analyses with very different model assumptions. Just in case a reader is wondering where error bars/estimates are, this would address it clearly.

*Due to word count restrictions we address this concern solely in the methods section. We now note the following on line 243: In efforts to gauge the sensitivity of the ROI results to key model assumptions,* ***and given the inability to access the raw data presented in Cawley et al for producing confidence intervals around the estimates****, we present a series of one-way sensitivity analyses. The key assumptions are…*

-Figure 1: the exclusion says BMI<26, but the authors indicate in the methods that exclusion was BMI<=25

*Thank you for pointing this out and we apologize for the oversight. We now fix line 100 to read: “Individuals who are employed by one of the 150 collective bargaining units and eligible for health benefits under the NYC Health Benefits Program and with a BMI of 26 kg/m^2^ or more were eligible for WW program enrollment.”*

**Reviewer #4: Comments to the Author**I commend the authors as they have made quite a substantial effort to address my previous comments and those of the other reviewer. Their responses are thorough and enhance the reader’s understanding of their methods. The authors have also made additional efforts to tone down their clams and discuss the limitations of their study.  I appreciate the sensitivity analysis as I think it allows the reader to assess the impact of the author’s assumptions and gives more validity to the work.

*Thank you for the positive comments. We greatly appreciate it.*

There are just a couple points that I feel need to be addressed. A couple of specific points,

1) The authors have done well to state that their savings calculations are predicted and not actually realized savings.  However, on line 137 “Annual net saving for NYC and ROI” are listed as calculated measures.  It should be made clear that both values are predictions.

*Thank you for pointing this out. We have changed the text to “(vi) Predicted annual net savings for NYC and predicted ROI.”*

2) On line 204-205 the authors state that “For the less than 1% of participants with starting BMI values above 45 (n=858) we conservatively recoded the BMI to 45.”  The n of 858 and less than 1% of participants does not appear to match.  I am assuming one of these numbers is an error.

*Thank you for pointing this out. 858 participants is correct but our math was wrong. The sentence now reads: “For the* ***5.7%*** *of participants with starting BMI values above 45 kg/m^2^ (n=858) we conservatively recoded their BMI to 45 kg/m^2^ and assigned savings as reported in Cawley et al. based on the amount of weight lost.”*

3) On line 252 the authors state that costs are 50% of the Cawley et al. Table 1.  Since the values in Table 1 are reductions in cost I believe the authors should refer to these values as savings rather than costs.

*Thanks for pointing this out. We now note:“Medical expenditure savings are 50% of what is reported in Cawley et al. Table 1.”*

4) The value of gross predicted savings to NYC of $2,999,858 does not account for the increased cost of weight gain and does not match the total gross savings listed in tables 2, 3, and 4 of $2,522,529. The authors also use this value of $2,999,858 for total gross savings in the base case in their sensitivity analysis.  It would be more appropriate to use $2,522,529 as the base case in this analysis.

*The value of gross predicted savings to NYC of $2,999,858 is the total gross predicted savings for employees and dependents combined and it does take into account the increased cost of weight gain. If you look at Tables 2,3, and 4, the gross predicted savings are separately listed as $2,522,529 for employees and $477,329 for dependents. These sums up to a total gross predicted savings to NYC of $2,999,858. Hope this clarifies why we use total gross predicted savings of $2,999,858 as the base case in this analysis.*

5) On line 399-400 the authors state that “This results because, among the 19% who generated savings, savings were large.”  It is unclear where the 19% comes from as table 4 shows that 35% of participants had weight loss that attributed to savings.

*We agree this point was not clear. We have now added the following at line 347 to clarify: “Table 4 consists of those eligible to generate savings. However, even if weight loss exceeds 2.5%, eligible participants may not generate savings. This would occur if they do not meet other criteria, including participation duration less than 90 days and/or enrollment BMI less than 30 kg/m^2^. Although Table 4 shows that 35% of participants had weight losses of 2.5% or more, only 2,426 participants (19%) out of those eligible to contribute to savings actually did so.” Hope this is now clear. Thanks again for the feedback.*
